# CD8 T cells promote heart failure progression in mice with preexisting left ventricular dysfunction

**DOI:** 10.3389/fimmu.2024.1472133

**Published:** 2024-09-11

**Authors:** Dongzhi Wang, Xinyu Weng, Wenhui Yue, Linlin Shang, Yidong Wei, John S. Clemmer, Yawei Xu, Yingjie Chen

**Affiliations:** ^1^ Department of Physiology and Biophysics, University of Mississippi Medical Center, Jackson, MS, United States; ^2^ Department of Cardiology, Tenth People’s Hospital, Tongji University, Shanghai, China; ^3^ Department of Cardiology, Zhongshan Hospital, Fudan University, Shanghai, China; ^4^ Lillehei Heart Institute, University of Minnesota Medical School, Minneapolis, MN, United States; ^5^ Department of Clinical Pharmacy, School of Pharmacy, Nanjing Medical University, Nanjing, China

**Keywords:** heart failure, CD8, regulatory T cell, lung, inflammation, fibrosis

## Abstract

**Introduction:**

Even under the standard medical care, patients with left ventricular (LV) failure or heart failure (HF) often progress to pulmonary hypertension and right ventricular (RV) hypertrophy. We previously showed that inflammation and regulatory T cells (Tregs) modulate HF progression in mice with preexisting LV failure. The main objective of this study is to determine the role of CD8^+^ T cells in modulating LV failure and the consequent pulmonary inflammation and RV hypertrophy in mice with preexisting LV failure.

**Methods:**

Mice with LV failure produced by transverse aortic constriction (TAC) were randomized to depletion of cytotoxic CD8^+^ T cells, Tregs, or both using specific blocking antibodies. Cardiac function, lung inflammation, fibrosis, vascular remodeling, and right ventricular remodeling were determined.

**Results:**

LV failure caused pulmonary inflammation, fibrosis, vascular remodeling, and RV hypertrophy. Depletion of CD8^+^ T cells significantly attenuated above changes in mice with preexisting LV failure. LV failure was associated with increased CD4^+^ and CD8^+^ T cell activation, and increased ratios of activated T cells to Tregs. Treg depletion exacerbated lung inflammation and HF progression, as well as lung CD4^+^ and CD8^+^ T cell infiltration and activation in HF mice. However, CD8^+^ T cells depletion rescue these mice from exacerbated lung inflammation and RV hypertrophy after Treg depletion.

**Discussion:**

Our findings demonstrate an important role of CD8^+^ T cells in promoting pulmonary inflammation and RV hypertrophy in mice with preexisting LV failure. Depletion of CD8^+^ T cells also rescued HF mice from the exacerbated HF progression by Treg depletion.

## Introduction

Heart failure (HF) or left ventricular (LV) failure is a condition in which the left ventricle is unable to pump out sufficient oxygen-rich blood into the systemic circulation to meet the body’s needs. Chronic hypertension can cause hypertensive cardiac disease and LV failure, which can further progress to severe lung vascular remodeling and fibrosis, and subsequent right ventricular (RV) hypertrophy and/or RV failure in experimental animals and in HF patients (1, 2). HF patient with pulmonary hypertension and RV hypertrophy significantly have a worse clinical outcome (3–5). However, most HF studies are focused on HF development, the process from normal heart to LV failure, while the underlying mechanism(s) responsible for HF progression and consequent pulmonary remodeling and RV hypertrophy, a process we define as HF progression, are still poorly understood.

Our previous studies demonstrate that end-stage HF causes profound lung inflammation (6–8) and an increase in lung activated CD4^+^ and CD8^+^ effector memory T cells (Tem) (9), suggesting that T cells might contribute to HF progression and consequent pulmonary remodeling and RV hypertrophy and/or failure. Our previous studies also demonstrated that inhibition of the inflammatory response by induction of endogenous T regulatory cells (Tregs) attenuated the transition from LV failure to RV hypertrophy in mice with existing LV failure produced by chronic systolic overload (8), while Pm2.5-induced lung inflammation promoted the transition from LV failure to RV hypertrophy (10). In addition, studies have demonstrated that CD4^+^ T cells and NK cells contribute to transverse aortic constriction (TAC)-induced LV inflammation, fibrosis, and dysfunction (11–13). We have also demonstrated that inhibition of CD4^+^ and CD8^+^ T cell activation by CD28 or B7 knockout and depletion of CD11c^+^ dendritic cells were effective in attenuating HF development (14).

Cytotoxic CD8^+^ T cells play an important role in modulating cardiac and pulmonary inflammatory responses during infection, autoimmune diseases such as autoimmune cardiomyopathy or myocarditis, and ischemic cardiac remodeling (15–18). Given that T cell activation significantly contributes to the development of HF (9, 11, 19–21), and the important role of CD8^+^ T cells in modulating inflammatory responses in various diseases, we investigated the role of CD8^+^ T cells in HF progression in mice with preexisting LV failure, and their role in the exacerbated LV dysfunction in mice after depletion of Tregs.

## Methods

Detailed methods are available in the online-only Data Supplement.

### Experimental protocol

Male Balb/c mice (~7 weeks of age) were subjected to a Transverse Aortic Constriction (TAC) (22), a commonly used experimental model to mimic clinical systemic hypertension or aortic stenosis. Two weeks after TAC, LV ejection fraction (EF) of these Balb/c mice was determined and animals were further divided into two experimental groups to assure similar initial LV dysfunction in these mice. The mice were then enrolled in treatment groups as described in the results and figures. Samples were collected 2 weeks after initiation of the treatments, at a time when the untreated mice had an average LV ejection fraction of ~35%.

### *In vivo* antibody treatment

CD8 and Treg depletion was achieved according to protocol developed by Ueha et al. with minor changes (23). Briefly, CD8 depletion was achieved by intra-peritoneal injection of anti-CD8 monoclonal antibody clone YTS 169.4 (BioXcell) in a dose of 200 µg/mouse twice a week for two weeks. Treg depletion was achieved by administration of anti-CD25 monoclonal antibody (mAb) clone PC61 (Biolegend) in a dose of 100 µg/mouse/week starting at two weeks after TAC (23). Control groups were treated with nonspecific isotype matched IgG. Peripheral blood was collected from retinal vein. Heart, lung and spleen were collected for flow cytometry, protein and RNA analysis or histological analysis. The studies were approved by the IACUC of Tongji University.

### Echocardiography and evaluation of RV hemodynamics

Mouse echocardiography image was obtained with a Visual sonics high-resolution Vevo 2100 system under the condition of anesthesia with 1% to 2% isoflurane (24, 25). Two-dimensional guided M-mode echocardiograms were acquired from short-axis view at the level of the maximum left ventricular (LV) diameter. Measurements of LV wall thickness at the end of systole and diastole were taken from the M-mode images. LV fractional shortening (FS) and ejection fraction (EF) were then calculated based on the measured dimensions of the ventricle. Right ventricular systolic pressure (RVSP) was measured by closed-chest RV catheterization. Briefly, after anesthesia with 1% isoflurane, the 1.2F Rodent PV Catheter (Transonic Systems Inc, Ithaca, NY) were placed into the right external jugular vein of mice. Data were analyzed with the analysis software Labscribe2 (iWorx, Dover, NH).

### Flow cytometric analysis

Single cells were dissociated from lungs and spleens. Flow cytometric analysis was performed as previously described (8). Lung tissues were cut into small pieces, and enzymatically digested in 5 ml of digestion buffer (HBSS without Ca^2+^/Mg^2+^ (Life technologies), 1 mg/ml collagenase (Roche)) at 37°C for 30 min with agitating, and then filtered with a 100μm strainer. Individual spleen was collected, cut into small pieces, and passed through a 100 μm strainer. Single cell suspensions were obtained via washing with 10 ml cold buffer (PBS + 0.5%BSA + 2 mM EDTA). Red Blood Cell Lysing Buffer (Sigma) were used to remove the erythrocytes. Cells were counted with a hemocytometer. Single cells suspensions were pre-incubated with anti-mouse CD16/32 (clone 2.4G2) antibody to prevent non-specific binding of antibodies to FcRγ, followed by multi-staining with fluorescence directly conjugated primary antibodies (**Supplementary Table S2**). Samples were subjected to FACS analysis (BD Biosciences). Data were analyzed by FlowJo_V10 (FlowJo, OR) software. Values represent the mean of five independent experiments.

### Statistics

A normality test (Shapiro-Wilk) provided by SPSS was used to determine whether data were normally distributed. If data were normally distributed, the data are presented as mean ± SEM. A Student’s t-test was used to test for differences between 2 groups. A one-way ANOVA followed by a Bonferroni correction *post-hoc* test was used to test for differences among groups greater than 2. If mouse physiological data were not normally distributed, a non-parametric test (Mann-Whitney or Kruskal-Wallis) followed by a Bonferroni *post hoc* correction was performed. All pairwise p-values are two-sided. The null hypothesis was rejected at P < 0.05.

## Results

### LV failure caused lung CD8^+^ T cell infiltration and increased CD8^+^ activation

**Figure 1:**
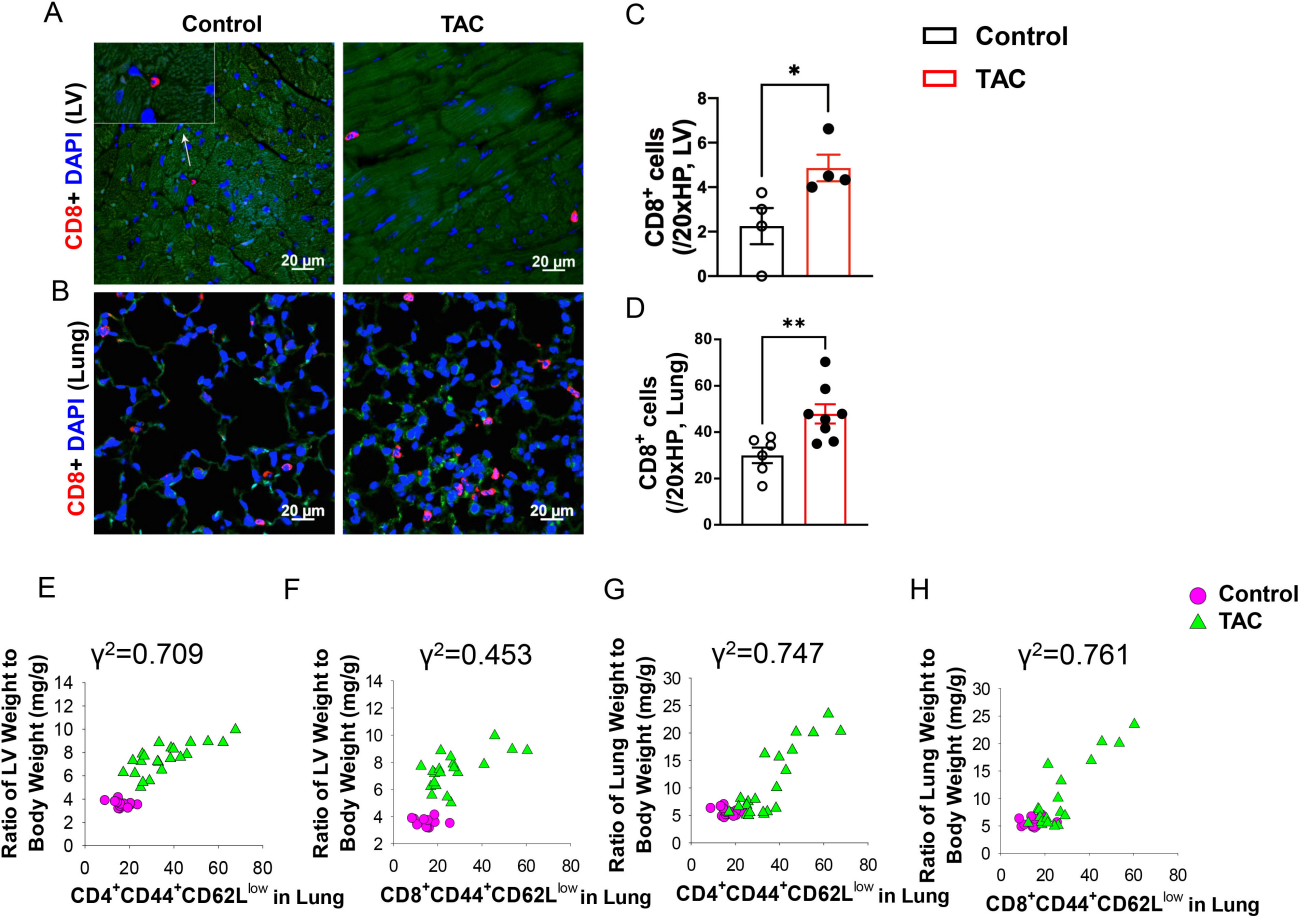
Increased CD8^+^ T cell infiltration and activation in lungs and hearts of heart failure mice. **(A, B)**, Immunofluorescence of CD8^+^ cells in the left ventricle (LV) and lung of mice after TAC. Red color represents CD8^+^ cells, blue color shows DAPI, green color represents autofluorescence. HP, high power objective (20x). **(C, D)**, Quantitative data represent the number of CD8^+^ T cells in the LV and lung. **(E–H)**, Correlations between LV weight, lung weight and active CD4^+^CD44^+^CD62L^-^ and CD8^+^CD44^+^CD62L^-^. Data represent mean ± SEM. *P<0.05; **P<0.01.

As shown in **Figure 1**, both LV and pulmonary CD8^+^ T cell infiltration was significantly increased in HF mice, and pulmonary CD8^+^ T cell infiltration was more dramatic as compared to LV CD8^+^ T cell infiltration (**Figures 1A–D**). Flow cytometry showed that the percentages of lung CD8^+^ Tem (CD8^+^CD44^+^CD62L^-^) and CD4^+^ Tem (CD4^+^CD44^+^CD62L^-^) were significantly increased after HF, while the percentages of lung naïve T cells (CD8^+^CD44^-^CD62L^+^ or CD4^+^CD44^-^CD62L^+^), which are characterized as high expression of CD62L and absence of activation marker CD44, were significantly reduced in HF mice (**Supplementary Figures S1A–D**). Interestingly, the increases in the percentage of lung CD4^+^ Tem and CD8^+^ Tem were significantly correlated with the ratio of LV weight to bodyweight (an index of LV hypertrophy) and the ratio of lung weight to body weight (an objective index of LV failure) (**Figures 1E–H**).

### Anti-CD8 mAb effectively reduced systemic and lung CD8^+^ T cells in HF mice

**Figure 2:**
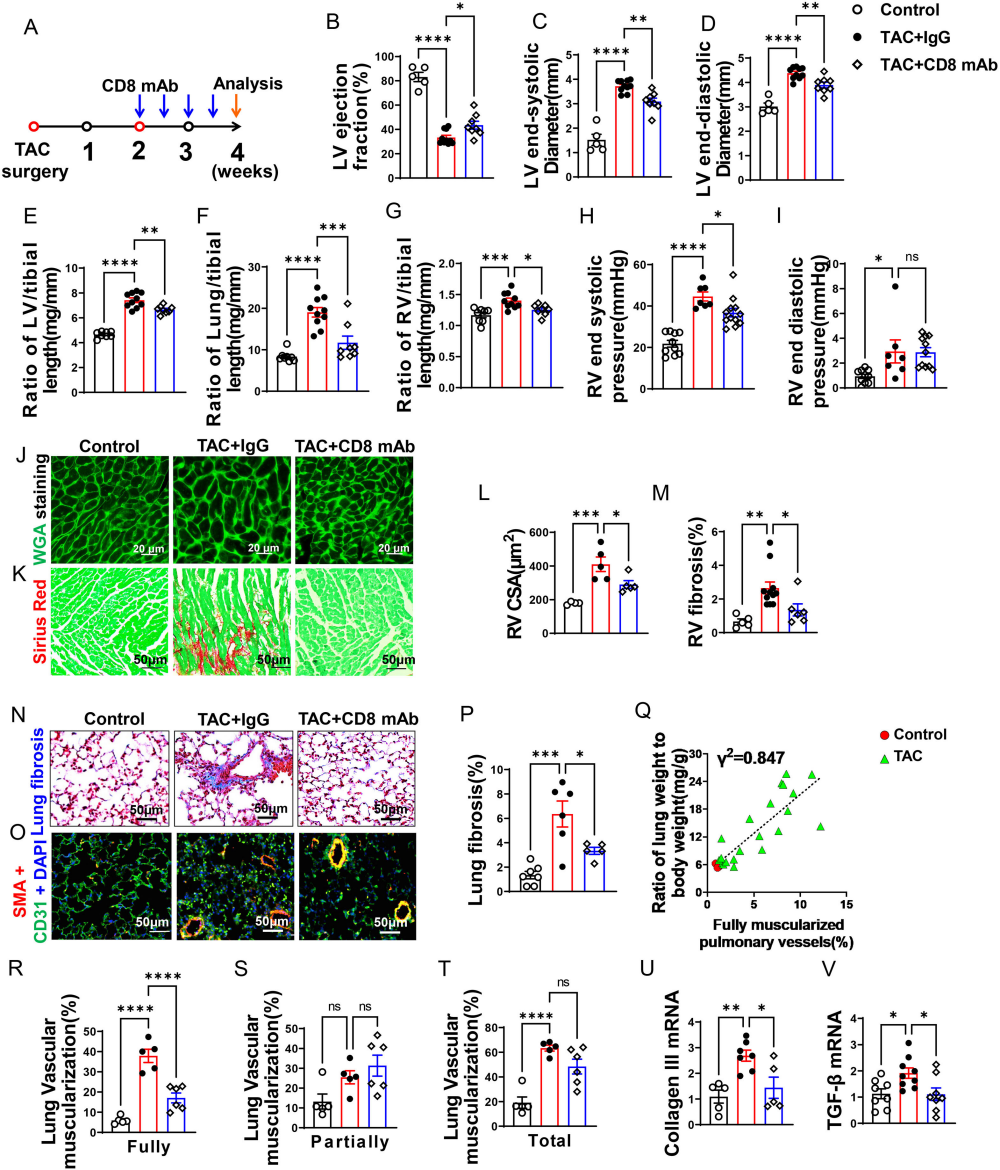
CD8^+^ T cell depletion attenuates transverse aortic constriction (TAC)-induced heart failure and pulmonary remodeling. **(A)**, Schematic diagram of the timeline for surgery and antibody dosage. Treatment was started two weeks after TAC surgery, Balb/C mice were randomly divided into two groups, and then injected i.p. with CD8 mAb (200ug/mouse) or isotype control antibody separately at days 4 and 7 for two weeks. **(B–D)**, Echocardiographic evaluation of cardiac function. **(B)**, Left ventricular ejection fraction (%). **(C)**, Left ventricular end-diastolic diameter (mm). **(D)**, Left ventricular end-systolic diameter (mm). **(E–G)**, The ratio of LV, lung and RV weight to tibial length of mice. **(H, I)**, hemodynamic evaluation of RV function. **(H)**, RV end systolic pressure (mmHg). **(I)**, RV end diastolic pressure (mmHg). **(J, (K)**, Representative images of wheat germ agglutinin (WGA) staining for detection of myocyte size of RV and Sirius red/Fast green staining for detection of fibrosis in RV. **(L, M)**, Quantitative data of WGA staining for detection of myocyte cross-sectional area (CSA) of RV and Sirius red/Fast green staining for detection of fibrosis in RV. **(N)**, Representative images of Masson trichrome staining for quantification of fibrotic areas in lung. **(O)**, Representative images of double immunostaining of the endothelial cell marker CD31 (green) and the smooth muscle cell marker α-actin (red) (SMA) in lung; nuclei were stained by DAPI (blue). **(P)**, Quantitative data of lung fibrosis. **(Q)**. Correlations between lung weight and fully muscularized pulmonary vessels (%). **(R–T)**, Quantitative data represent the muscularization of lung arterioles. **(U, V)**, RT-qPCR of analyzing the expression of Collagen III and TGF-β in lung. Data represent mean ± SEM. *P<0.05; **P<0.01; ***P<0.001, ns: no significance.

To determine whether CD8^+^ T cells contribute to the transition from LV failure to RV hypertrophy in mice with existing LV failure produced by TAC, anti-CD8 mAb treatment was used as described in **Figure 2A**. As shown in **Supplementary Figures S2A–C**, anti-CD8 mAb treatment was effective in reducing systemic and tissue CD8^+^ T cells as evidenced by a reduction of total CD8^+^ T cells 79% in blood, 70% in spleen and 60% in lung, respectively. The HF mice treated with anti-CD8 mAb appeared more physical active as compared to HF mice treated with control IgG. The HF-induced bodyweight reduction was significantly attenuated by depletion of CD8^+^ T cells (**Supplementary Figure S2D**), suggesting an overall reduction in cardiac cachexia in HF mice with CD8 depletion.

### Depletion of CD8^+^ T cells significantly attenuated the reduction of LV ejection fraction, increase of lung weight, and RV hypertrophy after LV failure

As presented in **Figures 2B–D**, echocardiographic measurements showed that TAC caused LV dysfunction in IgG treated mice as evidenced by the significantly reduced LV ejection fraction, increased LV end-systolic diameter (LVESD), and increased LV end-diastolic diameter (LVEDD) as compared with the sham group. Depletion of CD8^+^ T cells was sufficient and effective in attenuating further reduction of LV function in these mice as indicated by a significantly smaller reduction of LV ejection fraction, and a significantly smaller increase of LVESD and LVEDD as compared with HF mice treated with control IgG (**Figures 2B–D**). The ratios of LV, lung and RV weight to tibial length were increased 1.6-fold, 2.3-fold, and 1.2-fold in IgG treated HF mice as compared with the sham group (**Figures 2E–G** and **Supplementary Table S3**). The LV weight and its ratio to tibial length were moderately reduced after depletion of CD8^+^ T cells (**Figure 2E** and **Supplementary Table S3**). On the other hand, lung weight, RV weight and their ratios to tibial length were remarkably attenuated by depletion of CD8^+^ T cells in mice with existing LV failure (**Figures 2F, G**; **Supplementary Table S3**). As presented in **Figures 2H, I**, RV end-systolic pressure increased by 1.7-fold, and diastolic pressure increased by 3.1-fold in HF mice as compared with the sham mice. Depletion of CD8^+^ T cells significantly attenuated LV failure-induced RV end-systolic pressure. Depletion of CD8^+^ T cells did not significantly affect RV diastolic pressure in HF mice treated with IgG. Both RV dp/dtmin and dp/dtmax showed an increase post-TAC, but CD8^+^ T cell depletion did not significantly affect above changes (**Supplementary Figures S2E, F**). Nevertheless, histological staining further demonstrated that depletion of CD8^+^ T cells significantly attenuated RV cardiomyocyte hypertrophy (**Figures 2J, L**) and RV fibrosis in mice with existing LV failure produced by TAC (**Figures 2K, M**).

### CD8^+^ T cell depletion attenuated pulmonary vascular muscularization and lung fibrosis in mice with existing LV failure

HF-induced increase of lung weight and pulmonary hypertension are associated with lung vascular muscularization and fibrosis (6). We determined lung fibrosis and lung vessel muscularization in HF mice with or without CD8 depletion. Masson’s trichrome stain showed that lung fibrosis increased 4.7-fold in HF mice as compared to that in control mice, and depletion of CD8^+^ T cells significantly attenuated lung fibrosis in mice with existing LV failure (**Figures 2N, P**). In addition, lung fully muscularized vessels and total muscularized vessels (including both fully and partially muscularized vessels) were all significantly increased in HF mice as compared to that in control sham mice (**Figures 2O, R, T**). There was a trend of increasing partially muscularized vessels, although no statistical difference has been observed (**Figure 2S**). Depletion of CD8^+^ T cells significantly attenuated the lung vessel muscularization in mice as evidenced by reducing the number of muscularized vessels (including both fully and total muscularized vessels) in these mice (**Figures 2O, R, T**). The percentage of fully muscularized vessels was significantly correlated to the increase of lung weight or its ratio to body weight (**Figure 2Q**), suggesting that lung weight represented parenchymal hypertrophy. Moreover, depletion of CD8^+^ T cells significantly attenuated lung collagen III content, and mRNA of transforming growth factor-β (TGF-β), a pro-fibrotic molecule (**Figures 2U, V**).

### Depletion of CD8^+^ T cells attenuated lung leukocyte infiltration and the increase of lung CD4^+^ Tem in mice with existing LV failure

**Figure 3:**
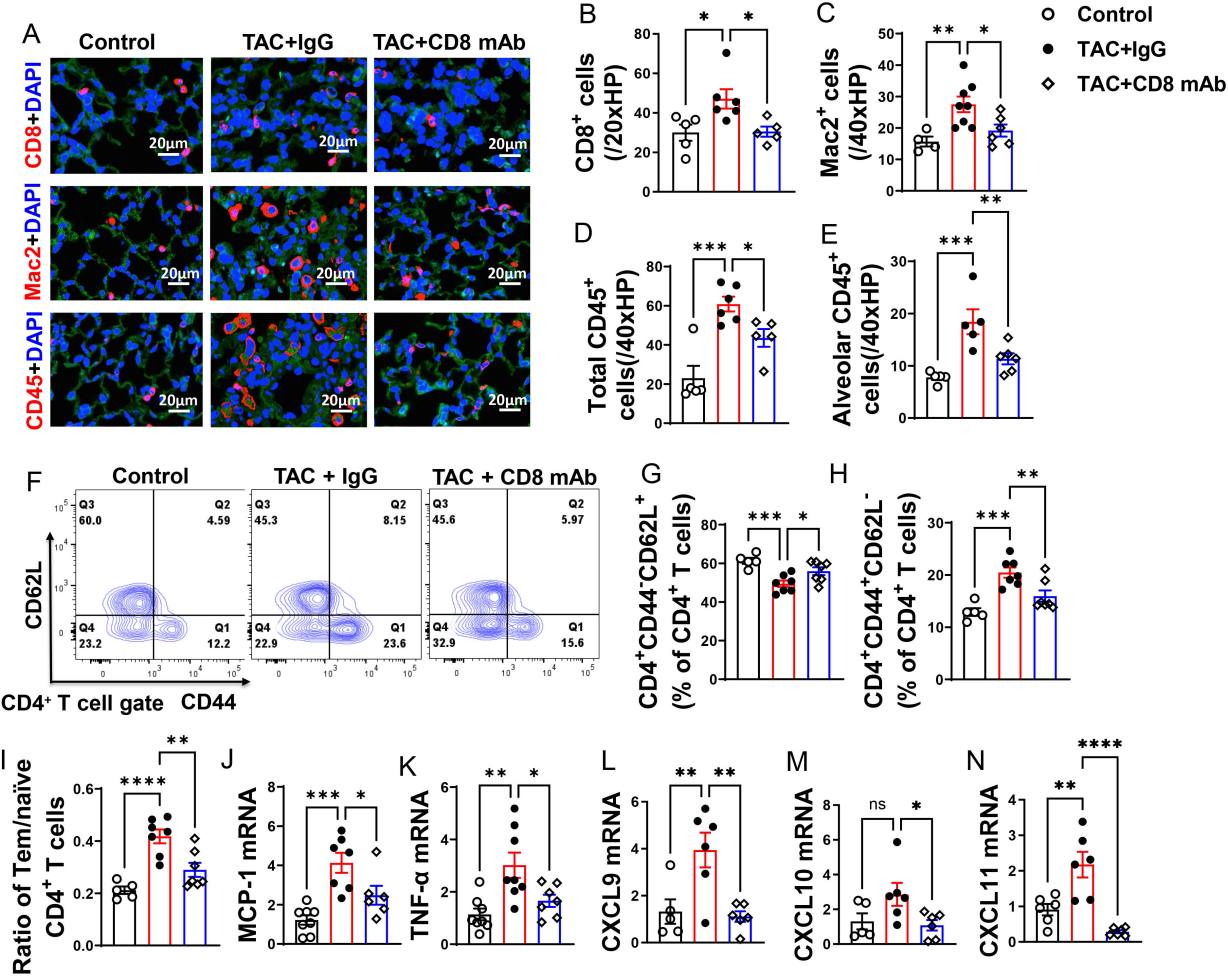
Depletion of CD8^+^ T cells attenuates lung leukocyte infiltration and inflammation in mice with existing LV failure. **(A)**, Representative images of immunofluorescence staining of CD8 for cytotoxic T cell marker, Mac2 for macrophage marker, and CD45 for leukocyte marker. **(B–D)**, Quantitative data represents total number of CD8^+^, Mac2^+^ and CD45^+^ cells in lung. **(E)**, Quantitative data of CD45^+^ leukocytes in alveoli. **(F)**, Flow cytometry characterization of CD4^+^ T cells subsets in lung after CD8 depletion. **(G, H)**, Percentage of CD4^+^CD44^-^CD62L^+^ naïve T cells and CD4^+^CD44^+^CD62L^-^ active T cells in lung. **(I)**, Ratio of Tem to naïve T cells. **(J–N)**, mRNA levels of MCP-1, TNF-α, CXCL9, CXCL10, CXCL11 in lungs. *P<0.05; **P<0.01; ***P<0.001, ns: no significance.

We found that lung CD8^+^ T cells, macrophages, and CD45^+^ leukocytes were markedly increased in HF mice, but depletion of CD8^+^ T cells 2 weeks after TAC significantly attenuated the lung leukocyte infiltration in the lungs (**Figures 3A–D**). Moreover, the number of lung alveolar CD45^+^ leukocytes was increased 4.03-fold in HF mice, and depletion of CD8^+^ T cells significantly attenuated lung alveolar CD45^+^ leukocyte infiltration by 46% as compared with IgG treated HF mice (**Figure 3E**). Furthermore, we found HF caused increases of the average cell size of CD45^+^ leukocytes and macrophages, and depletion of CD8^+^ T cells significantly attenuated the average cell size of CD45^+^ leukocytes and macrophages in HF mice (**Supplementary Figures S2G, H**).

Since CD4^+^ T cells and activated T cells regulate tissue inflammation in several immune mediated conditions, we determined the activation status of CD4^+^ T cell subsets in lung (**Figures 3F–I**), blood and spleen (**Supplementary Figures S3A–I**). As anticipated, IgG treated HF mice showed a 1.5-fold increase of the percentage of lung CD4^+^ Tem, a 16% decrease of the percentage of lung naïve T cells, and a 3.6-fold increase in the ratio of lung CD4^+^ Tem to naïve T cells as compared with the data of the sham group (**Figures 3G–I**). Depletion of CD8^+^ T cells significantly attenuated the percentage of lung Tem and the ratio of lung Tem to naïve T cells in HF mice (**Figures 3H, I**). Depletion of CD8^+^ T cells also abolished the reduction of the percentage of lung naive T cells as compared with the sham group (**Figure 3G**). Lung central memory CD4^+^CD44^+^CD62L^+^ T cells and the CD4^+^CD44^-^CD62L^-^ T cell subsets were not significantly altered after HF, regardless of CD8 depletion (**Supplementary Figures S2I, J**).

In contrast to the notable increase of lung Tem and the ratio of Tem to naïve T cells, blood Tem, naïve, central memory, and CD4^+^CD44^-^CD62L^-^ T cells remained unchanged in HF mice with or without depletion of CD8^+^ T cells (**Supplementary Figures S3A–D**). Similarly, the ratio of blood Tem to naïve T cells also showed no significant change (**Supplementary Figure S3E**). The spleen CD4 Tem, naïve, central memory and CD4^+^CD44^-^CD62L^-^ T cells were mostly unaffected after HF with or without depletion of CD8^+^ T cells (**Supplementary Figures S3F–I**). These findings indicate that the Tem accumulation in HF is primarily specific to the lung.

### Depletion of CD8^+^ T cells significantly attenuated lung pro-inflammatory cytokine production in mice with existing LV failure

We observed a significant increase in lung mRNA levels of pro-inflammatory cytokines, including monocyte chemoattractant protein-1 (MCP-1) and tumor necrosis factor α (TNF-α). Interestingly, depletion of CD8^+^ T cells led to a significant reduction in these pro-inflammatory cytokines in mice with established LV failure (**Figures 3J, K**).

The chemokines CXCL9, CXCL10, and CXCL11 are generally produced by activated macrophages and dendritic cells to facilitate CD8^+^ T cell infiltration to injured or tumor tissues. In our study, we observed a significant increase in lung mRNA levels of CXCL9 and CXCL11 following HF, while CXCL10 showed a minor increase trend that was not statistically significant (**Figures 3L–N**). Furthermore, depletion of CD8^+^ T cells attenuated the lung mRNA levels of CXCL9, CXCL10, and CXCL11(**Figures 3L–N**). Together, these data demonstrate that accumulation of cytotoxic CD8^+^ T cells in the lung of the HF mice may be partially attributed to the elevated chemokine CXCR9 and CXCR11.

### CD8^+^ T cell depletion had no effect on the percentage of Tregs in blood, lung and spleen in mice with existing LV failure

Since Tregs regulate inflammatory responses in various disease conditions (26), we further determined the potential effect of depletion of CD8^+^ T cells on Tregs in lung, peripheral blood and spleen. The percentage of lung CD4^+^CD25^+^Foxp3^+^ Tregs in CD4^+^ T cells was not significantly changed in HF mice with or without depletion of CD8^+^ T cells (**Supplementary Figures S4A, B**). As the expression of CD25 and Foxp3 contributes to the immune suppressive role of Tregs (27), we also determined the relative CD25 and Foxp3 expression in Tregs by calculating the relative Mean Fluorescence Intensity (MFI) of CD25 and Foxp3. The MFI of lung CD25 or Foxp3 in Tregs was also unchanged in HF mice with or without depletion of CD8^+^ T cells (**Supplementary Figures S4C, D**). MFI of CD25 or Foxp3 in Tregs was also unchanged in spleen or blood in HF mice with or without depletion of CD8^+^ T cells (**Supplementary Figures S4E–J**).

### CD25 mAb was effective in depletion of Tregs in mice with existing LV failure

**Figure 4:**
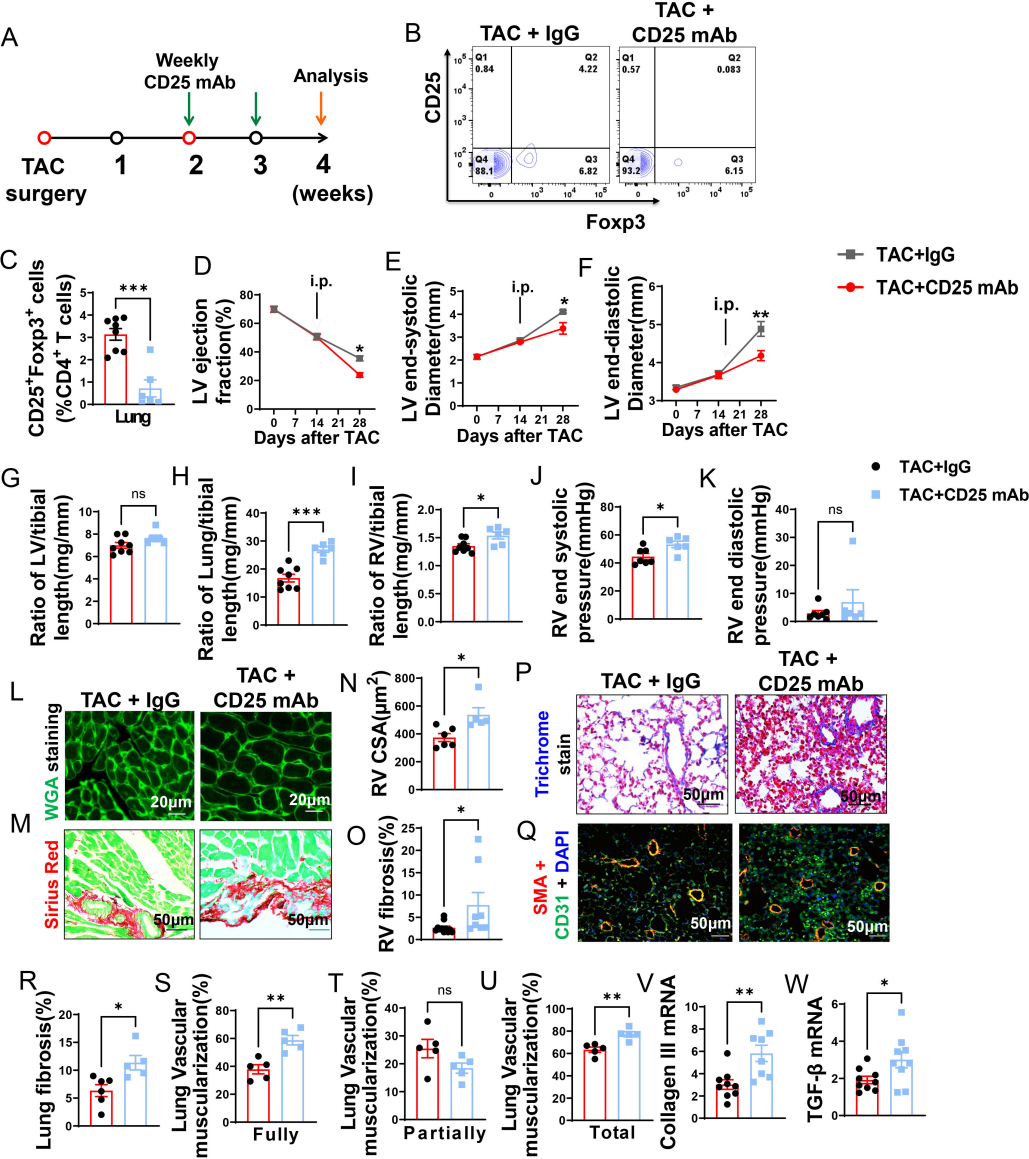
CD25 blockage exacerbates TAC induced LV failure, pulmonary remodeling and RV hypertrophy. **(A)**, Schematic of timeline for surgery and antibody dosage strategy. Treatment was started when LV ejection fraction **(E, F)** reached around 55% after TAC surgery, and then intraperitoneally injected with CD25 mAb or isotype control antibody once a week for two weeks. **(B)**, Representative flow cytometric plots in lung, gated on CD4^+^ T cells. **(C)**, The percentage of regulatory T cells (Tregs; CD25^+^Foxp3^+^) in lung. **(D–F)**, Echocardiographic evaluation of cardiac function. **(D)**, Left ventricular ejection fraction (%). **(E)**, Left ventricular end-diastolic diameter (mm). **(F)**, Left ventricular end-systolic diameter (mm). **(G–I)**, The ratio of LV, lung and RV weight to tibial length of mice. **(J)**, RV end systolic pressure (mmHg). **(K)**, RV end diastolic pressure (mmHg). **(L, M)**, Representative images of WGA staining for detection of myocyte size of RV and Sirius red/Fast green staining for detection of fibrosis in RV. **(N, O)**, Quantitative data of WGA staining for detection of myocyte size of RV and Sirius red/Fast green staining for detection of fibrosis in RV. **(P)**, Masson trichrome staining for quantification of fibrotic areas in lung. **(Q)**, Representative images of double immunostaining of the endothelial cell marker CD31 (green) and the smooth muscle cell marker α-actin (red) in lung; nuclei stained by DAPI (blue). **(R)**, Quantitative data of lung fibrosis. **(S–U)**, Quantitative data represents the muscularization of lung arterioles. **(V, W)**, mRNA expression levels of collagen III and TGF-β in the lungs. *P<0.05; **P<0.01; ***P<0.001, ns: no significance.

Even though Tregs were unchanged during depletion of CD8^+^ T cells, they may still play a role in HF progression. Treg depletion was performed in mice with existing LV failure. Briefly, anti-CD25 mAb clone PC61 was given to mice once a week (**Figure 4A**). The anti-CD25 mAb treatment was effective in reducing Tregs in lung (**Figures 4B, C**) and spleen (**Supplementary Figure S5E**). In addition, the percentage of CD25^+^ and Foxp3^+^ cells in CD4^+^ T cells and expression of CD25 in Tregs was significantly reduced in lung and spleen of HF mice after Treg depletion, while the expression of Foxp3 in the remaining Treg subset was largely unchanged (**Supplementary Figures S5A–I**).

### Treg depletion dramatically exacerbated LV failure, increase of lung weight, and RV hypertrophy in mice with existing LV failure

Echocardiographic measurements showed that Treg depletion exacerbated the loss of LV function, as indicated by significantly more reduction of LV EF and greater increases of LVESD and LVEDD as compared with HF mice treated with control IgG (**Figures 4D–F**).

Depletion of Tregs didn’t further exacerbate LV hypertrophy in HF mice as evidenced by the unchanged LV weight and its ratio to tibial length (**Figure 4G**; **Supplementary Table S4**). However, Treg depletion significantly exacerbated the increase of lung weight 1.83-fold (**Figure 4H**; **Supplementary Table S4**). In addition, Tregs depletion significantly exacerbated RV hypertrophy as evidenced by the significantly increased RV weight and its ratios to tibial length or bodyweight (**Figure 4I**; **Supplementary Table S4**). In **Figures 4J, K**, the evaluation of RV function revealed that depletion of Treg notably increased RV end-systolic pressure by 1.2-fold, along with a trend towards increased RV diastolic pressure, although this change did not reach statistical significance. Similarly, both RV dp/dt-minimal and dp/dt-maximal showed an increase after Treg depletion, but statistical significance was not detected (**Supplementary Figures S6A, B**). Histological staining showed that the exacerbated RV hypertrophy was the result of increased cardiomyocyte hypertrophy and RV fibrosis (**Figures 4L–O**).

### Treg depletion exacerbated lung fibrosis and vascular muscularization in mice with existing LV failure

As shown in **Figure 4P**, Masson’s trichrome stain showed that Treg depletion exacerbated lung fibrosis 1.4-fold in HF mice (**Figures 4P, R**). Treg depletion also significantly exacerbated lung vessel muscularization in HF mice, as evidenced by a significant ~1.6-fold increase of fully muscularized vessels, as well as increases of totally muscularized vessels in HF mice (**Figures 4Q, S, U**), Partially muscularized vessels were not significantly changed (**Figure 4T**). Treg depletion also exacerbated lung mRNA contents of collagen III and TGF-β in HF mice (**Figures 4V, W**).

### Treg depletion exacerbated lung leukocyte infiltration and pro-inflammatory cytokines in mice with existing LV failure

**Figure 5:**
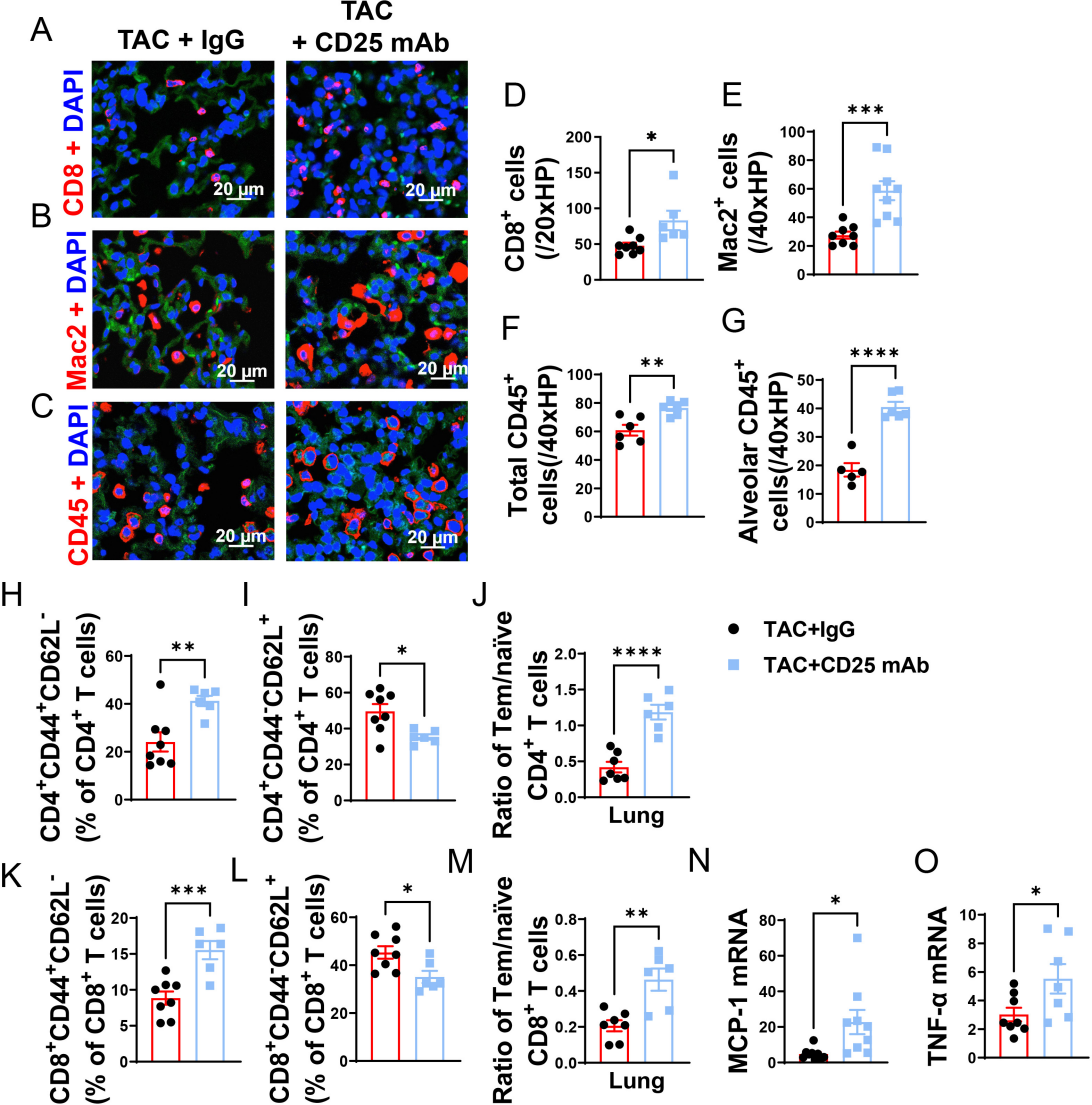
Treg Depletion exacerbates lung leukocyte infiltration and inflammation in mice with existing LV failure. **(A–C)**, Representative images of immunofluorescence staining of CD8 for cytotoxic T cell marker, Mac2 for macrophage marker and CD45 for leukocyte marker. **(D–F)**, Quantitative data represents total number of CD8^+^, Mac2^+^ and CD45^+^ cells in lung. **(G)** CD45^+^ cells in alveoli. **(H, I, K, L)**, Percentage of active CD4^+^ T cells, naïve CD4^+^ T cells, active CD4^+^ T cells and naïve CD8^+^ T cells in lung. **(J, M)**, Ratio of Tem to naïve T cells in CD4^+^ and CD8^+^ T-cell population of lungs. **(N, O)**, mRNA levels of MCP-1 and TNF-α in lung. *P<0.05; **P<0.01; ***P<0.001, ns: no significance.

We further determined lung CD8^+^ T cells, macrophages, and CD45^+^ leukocytes, as well as the mRNA contents of several inflammatory cytokines. As shown in **Figure 5**, Treg depletion significantly exacerbated infiltration of lung CD8^+^ T cells, macrophages, and CD45^+^ leukocytes in mice with existing LV failure (**Figures 5A–G**). Treg depletion also significantly exacerbated the infiltration of lung alveolar CD45^+^ cells in HF mice (**Figure 5G**). Moreover, the average cell size of lung CD45^+^ cells and macrophages were also significantly increased after Treg depletion in HF mice (**Supplementary Figures S6C, D**).

Given the capacity of Tregs in suppressing cytotoxic CD8 activity and autoimmunity, we further determined the impact of Treg depletion on lung inflammation and HF progression. As anticipated, Treg depletion significantly exacerbated the increase of CD4^+^ Tem (**Figure 5H**), the decrease of naïve CD4^+^ T cells, and the increase of the ratio of Tem to naïve CD4^+^ T cells (**Figures 5I, J**). Treg depletion did not significantly affect lung central memory CD4^+^CD44^+^CD62L^+^, and lung CD4^+^CD44^-^ CD62L^-^ T cells in HF mice (**Supplementary Figures S6E, F**). Treg depletion also significantly exacerbated the increase of CD8^+^ Tem, the decrease of naïve CD8^+^ T cells, and the increase of the ratio of Tem to naïve CD8^+^ T cells (**Figures 5K–M**). Treg depletion did not significantly affect lung central memory CD8^+^ and lung CD8^+^CD44^-^ CD62L^-^ T cells in HF mice (**Supplementary Figures S6G, H**). Furthermore, we found that spleen Tem, naïve and central memory T cells were largely unaffected in HF mice with or without Treg depletion (**Supplementary Figures S7A–I**). Treg depletion also didn’t affect the ratio of spleen CD4^+^ Tem to CD4^+^ naïve T cells and the ratio of spleen CD8^+^ Tem to naïve T cells in HF mice (**Supplementary Figures S7E, J**). Moreover, real-time PCR demonstrated that Treg depletion significantly increased lung mRNA contents MCP-1 and TNF-α as compared with IgG treated HF mice (**Figures 5N, O**). Taken together, these data indicated that Treg depletion enhanced LV dysfunction, pulmonary CD45^+^ leukocyte infiltration, T cell accumulation and activation, and RV hypertrophy in mice with preexisting LV failure.

### CD8 depletion rescued HF mice from Treg depletion-induced LV dysfunction, increase of lung weight, and RV hypertrophy in mice

**Figure 6:**
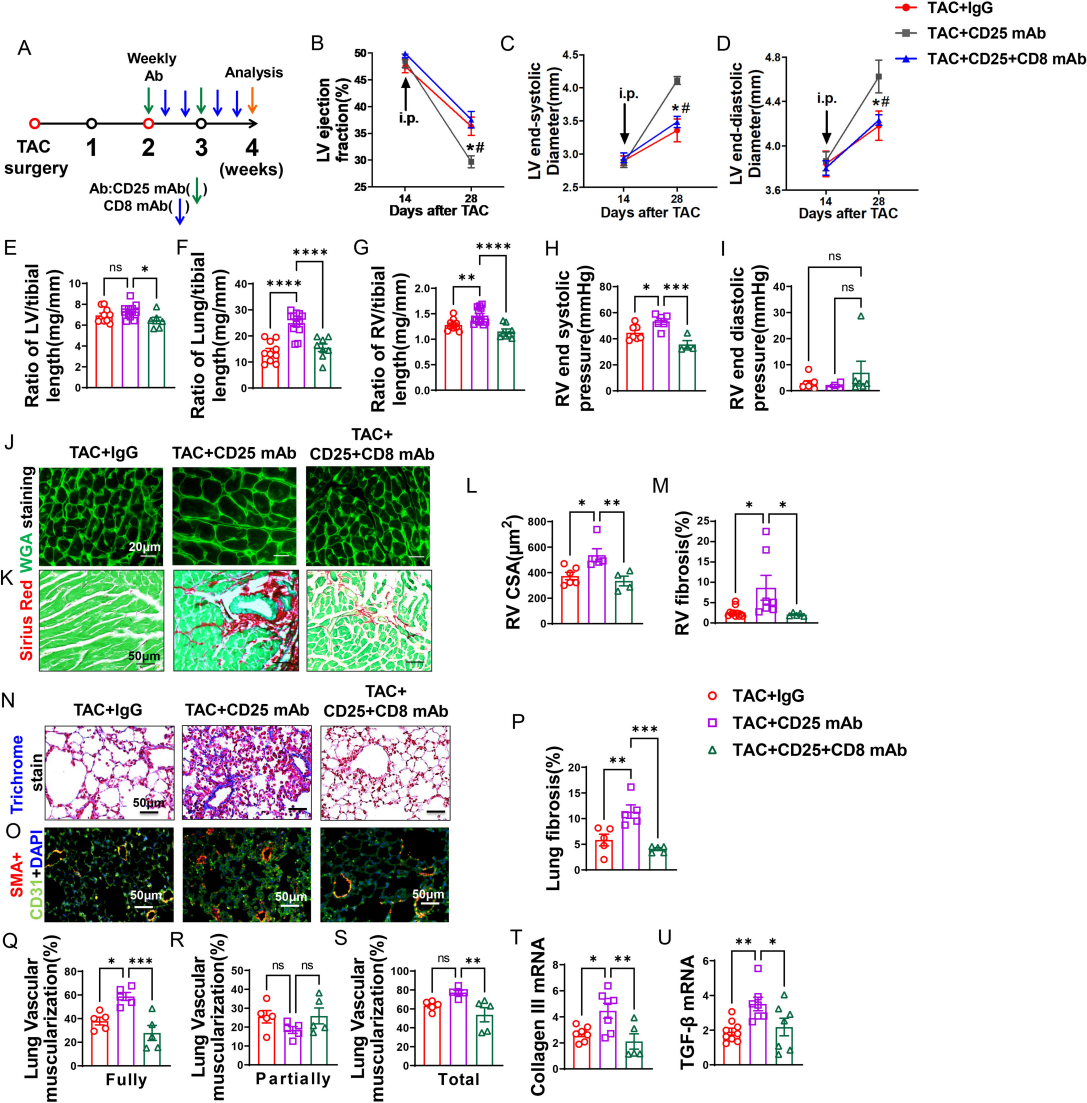
Anti-CD8 mAb rescued the aggravating effect of Treg depletion on TAC-induced LV failure, pulmonary congestion and RV hypertrophy. **(A)**, Schematic of timeline for surgery and antibody dosage strategy. Treatment was started when LV ejection fraction **(E, F)** reached around 55% after TAC surgery, and mice were then intraperitoneally injected with CD25 mAb once a week for two weeks with or without CD8 mAb (200ug/mouse) at days 4 and 7 for two weeks, or isotype control antibody. **(B–D)**, Echocardiographic evaluation of cardiac function. **(B)**, Left ventricular ejection fraction (%). **(C)**, Left ventricular end-diastolic diameter (mm). **(D)**, Left ventricular end-systolic diameter (mm). **(E–G)**, The ratio of LV, lung and RV weight to tibial length of mice. **(H)**, RV end systolic pressure (mmHg). **(I)**, RV end diastolic pressure (mmHg). **(J, K)**, Representative images of WGA staining for detection of myocyte size of RV and Sirius red/Fast green staining for detection of fibrosis in RV. **(L, M)**, Quantitative data of WGA staining for detection of myocyte size of RV and Sirius red/Fast green staining for detection of fibrosis in RV. **(N)**, Masson trichrome staining for quantification of fibrotic areas in lung. **(O)**, Representative images of double immunostaining of the endothelial cell marker CD31 (green) and the smooth muscle cell marker α-actin (red) in lung; nuclei were stained by DAPI (blue). **(P)**, Quantitative data of lung fibrosis. **(Q–S)**, Quantitative data represents the muscularization of lung arterioles. **(T, U)**, mRNA levels of collagen III and TGF-β in the lungs. *P<0.05; **P<0.01; ***P<0.001, ns: no significance.

Since Treg depletion profoundly exacerbated lung inflammation and vascular remodeling in mice with existing LV failure, we further determined whether depletion of CD8^+^ T cells could effectively rescue these HF mice. As illustrated in **Figure 6A**, depletion of CD8^+^ T cells was initiated at the same time as Treg depletion. Treg depletion resulted in similar ~80% reduction of Tregs in both lung and spleen in HF mice with or without CD8 depletion (**Supplementary Figures S8A, B, G**). Depletion of Tregs also resulted in significant reductions of CD25^+^ and Foxp3^+^ T cells in lung and spleen in HF mice, and the reduction was not affected by depletion of CD8^+^ T cells (**Supplementary Figures S8C, D, H, I**). Moreover, the expression of CD25 and Foxp3 in Tregs was also unchanged by depletion of CD8^+^ T cells (**Supplementary Figures S8E, F, J, K**).

Echocardiographic measurements showed that after Treg depletion mice exhibited a significant decrease of LV EF, an increase in LVESD, and an increase of LVEDD 4 weeks after TAC (**Figures 6B–D**). CD8 depletion totally rescued the mice from the exacerbated LV dysfunction caused by Treg depletion, as indicated by normalization of LV EF, LVESD and LVEDD in these mice (**Figures 6B–D**). In addition, the increases of the ratio of lung weight or RV weight to tibial length caused by Treg depletion were totally rescued in HF mice by depletion of CD8 (**Figures 6F, G**; **Supplementary Table S5**). Surprisingly, CD8 depletion attenuated the increasing of LV weight to tibial length in CD25 mAb treated HF mice (**Figure 6E**). Depletion of CD8^+^ T cells notably reduced the increase in RV end-systolic pressure caused by Treg depletion, with no significant change observed in RV diastolic pressure (**Figures 6H, I**). Additionally, both RV dp/dt-minimal and dp/dt-maximal showed an increase following CD8^+^ T cell depletion, although statistical significance was not achieved (data not shown). Histological staining further showed that CD8 deletion significantly attenuated the exacerbated RV cardiomyocyte hypertrophy and RV fibrosis in HF mice by Treg depletion (**Figures 6J–M**).

### Depletion of CD8^+^ T cells rescued HF mice from Treg depletion-induced lung fibrosis and vessel muscularization

Masson’s trichrome stain showed that Treg depletion exacerbated lung fibrosis, and depletion of CD8^+^ T cells rescued HF mice from the exacerbated lung fibrosis caused by Treg depletion (**Figures 6N, P**). In addition, both fully muscularized and total muscularized lung vessels were significantly increased in HF mice after Treg depletion, and depletion of CD8^+^ T cells rescued HF mice from Treg depletion-induced lung vessel muscularization (**Figures 6O, Q–S**). Moreover, depletion of CD8^+^ T cells significantly attenuated the increases of lung mRNA contents of collagen III and TGF-β (**Figures 6T, U**).

### Depletion of CD8^+^ T cells rescued HF mice from Treg depletion-induced lung inflammation

**Figure 7:**
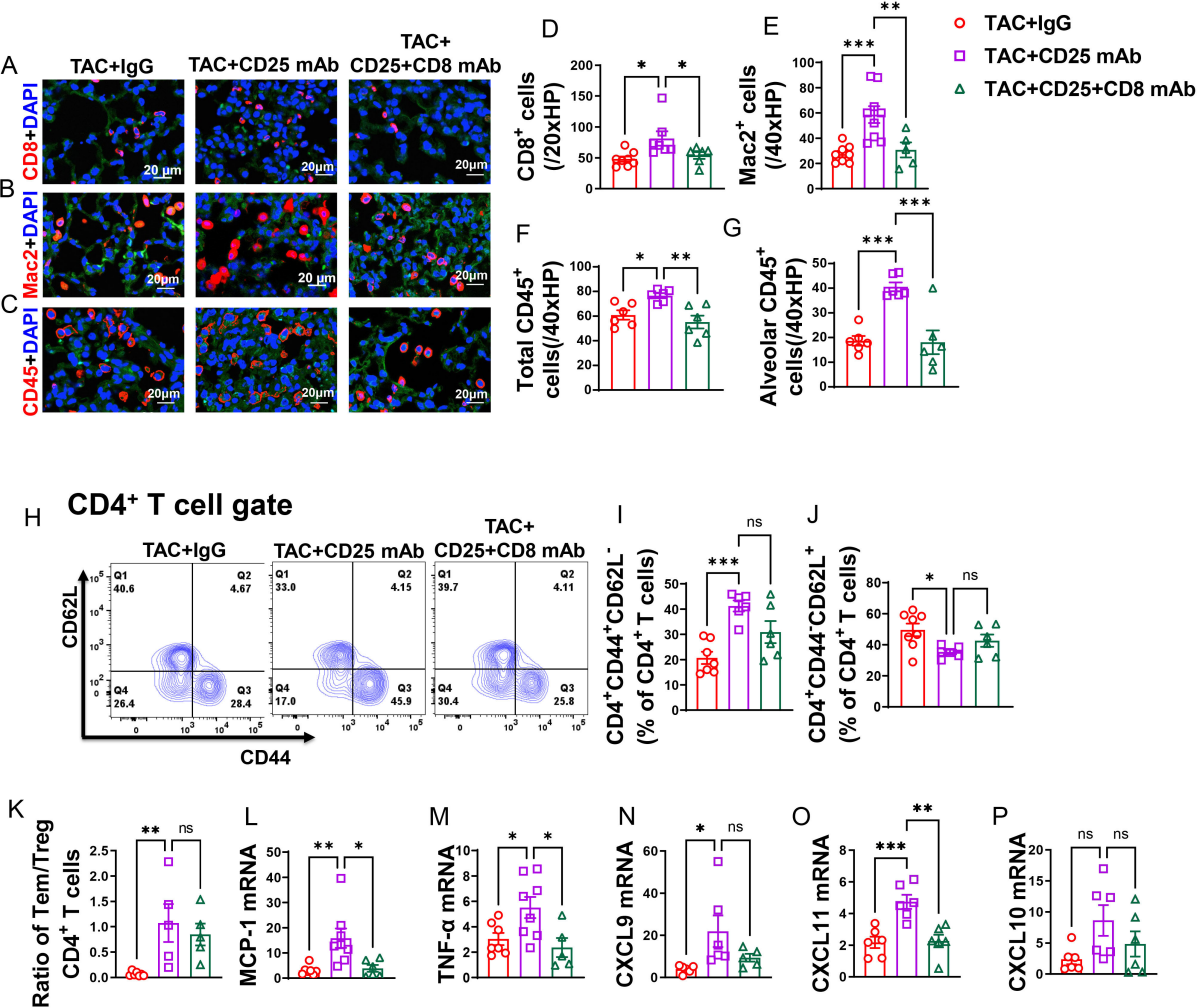
CD8^+^ T cell depletion rescued HF mice from Treg depletion-induced lung increased lung immune cell infiltration and inflammation. **(A–C)**, Representative images of immunofluorescence staining of CD8 for cytotoxic T cell marker, Mac2 for macrophage marker and CD45 for leukocyte marker. **(D–F)**, Quantitative data represents total number of CD8^+^, Mac2^+^ and CD45^+^ cells in lung. **(G)**, Quantitative data of CD45^+^ leukocyte in alveoli. **(H)**, Flow cytometry characterization of CD4^+^ T cells subsets. **(I, J)**, Percentage of active CD4^+^ T cells and naïve CD4^+^ T cells in lung. **(K)**, Ratio of Tem to naïve T cells and Treg in lungs. **(L–P)**, mRNA levels of MCP-1, TNF-α, CXCL9, CXCL11 and CXCL10 in lung. *P<0.05; **P<0.01; ***P<0.001, ns: no significance.

We demonstrated that lung CD8^+^, macrophage and CD45^+^ cell infiltration was enhanced after Treg depletion in HF mice (**Figures 7A–F**). Depletion of CD8^+^ T cells rescued HF mice from the exacerbated overall lung leukocyte infiltration (**Figure 7F**), and lung alveolar CD45^+^ leukocyte infiltration (**Figure 7G**). Depletion of CD8^+^ T cells also significantly attenuated the increased cell sizes of lung CD45^+^ leukocytes and macrophages induced by Treg depletion (**Supplementary Figures S9A, B**).

After Treg depletion in mice with HF, there was an observed increase in the percentage of lung CD4^+^ Tem (effector memory T cells), along with a further decrease in the percentage of naïve CD4^+^ T cells (**Figures 7H–J**). Additionally, the ratio of Tem to naïve CD4^+^ T cells significantly increased following Treg depletion (**Figure 7K**). Interestingly, while depletion of CD8^+^ T cells alongside Treg depletion appeared to rescue some of the alterations induced by Treg depletion alone in lung CD4^+^ Tem, naïve T cells, and the ratio of Tem to naïve T cells in HF mice, this effect did not reach statistical significance (**Figures 7I–K**). Lung central memory CD4^+^ T cells and CD4^+^CD44^-^CD62L^-^ T cells remained unaffected by Treg depletion in HF mice, regardless of CD8^+^ T cell depletion (**Supplementary Figures S9C, D**).

In contrast, the spleen CD4^+^ Tem, naïve CD4^+^ T cells, central memory CD4^+^ T cells, and CD4^+^CD44^-^CD62L^-^ T cells were largely unaffected by Treg depletion in HF mice, with or without concurrent CD8^+^ T cell depletion (**Supplementary Figures S9E–H**).

Additionally, it was noted that the lung mRNA levels of pro-inflammatory cytokines, such as MCP-1 and TNF-α, were elevated following Treg depletion (**Figures 7L, M**). Notably, depletion of CD8^+^ T cells was effective in mitigating the Treg depletion-induced increase in mRNA levels of MCP-1 and TNF-α (**Figures 7L, M**). Moreover, the chemokines CXCL9 and CXCL11 showed an increase after Treg depletion, and while depletion of CD8^+^ T cells alongside could notably alleviate the elevation in mRNA levels of CXCL11 and marginally decrease CXCL9 level, these effects did not reach statistical significance (**Figures 7N, O**). There was a trend toward increased mRNA levels of CXCL10 following Treg depletion; however, this trend did not reach statistical significance. Depletion of CD8^+^ T cells tended to mitigate this trend, although again, statistical significance was not achieved (**Figure 7P**).

## Discussion

We demonstrated for the first time that depletion of CD8^+^ T cells significantly attenuated TAC-induced increase of lung weight, lung leukocyte infiltration, lung fibrosis, lung vascular remodeling, and RV hypertrophy in mice with preexisting LV failure, indicating that CD8^+^ T cells promote HF development and progression. In addition, we demonstrated that end-stage HF was associated with an increased ratio of pulmonary Tem to Tregs, while systemic Treg depletion further exacerbated LV dysfunction, increase of lung weight, lung inflammation, lung fibrosis, lung vessel remodeling, and RV hypertrophy in mice with preexisting LV failure. Treg depletion also exacerbated the lung CD4^+^ and CD8^+^ T cell infiltration, and the percentage of Tem in these T cell subsets in HF mice. Moreover, we demonstrated that depletion of CD8^+^ T cells rescued HF mice from the exacerbated HF progression by Treg depletion, as evidenced by lesser degrees of LV dysfunction, lung inflammation, lung vascular remodeling, and RV hypertrophy. Together, these data demonstrate that CD8^+^ T cells play a critical role in HF development and progression in mice with or without Treg dysfunction, indicating that restraining the overreactive CD8^+^ T cells may be a potential therapeutic target for treating HF progression.

Our results demonstrated that the HF progression in mice with preexisting LV failure can be effectively attenuated or halted by inhibition of CD8^+^ T cells with untreated LV failure. These findings imply that acquired immunity by CD8^+^ T cells exerts an important role in cardiopulmonary inflammation and HF progression. While correcting the initial cause of LV failure is a primary goal for HF treatments, HF patients often don’t seek medical treatment until symptoms of LV failure occur. Therefore, the consequences of LV failure (such as lung vessel remodeling and RV hypertrophy or dysfunction) affect the subsequent clinical outcomes, including quality of life and life survival (2, 28).

Current treatments for patients with acute decompensated HF, such as diuretics and inotropic agents, have shown limited benefit on the long term of survival of HF patients. This may be due to the fact that these interventions have limited impact on HF-induced progression, such as the consequent pulmonary hypertension and RV hypertrophy after LV failure. Thus, therapeutic interventions that interrupt the secondary events of LV failure are still likely to be clinically important. For example, a previous study showed that LV failure caused profound global pulmonary vessel remodeling (29, 30), a finding consistent to our previous and current reports in experimental HF mice (6, 8, 9). Our findings suggest that new therapeutic methods even with no direct impact on LV contractility should be explored for HF treatment. These findings support the notion that effective therapy for advanced HF might require comprehensive treatment of both LV failure and the consequent lung remodeling, a concept proposed by us decades ago (6).

The increased cardiopulmonary CD45^+^ leukocytes, macrophages, and CD8^+^ T cells, as well as increased inflammatory cytokines (such as IL-1β and TNF-α), indicate tissue inflammation in these HF mice. While the mechanism for HF-induced pulmonary inflammation is unclear, the mechanical stress secondary to the increased pulmonary venous pressure is likely a major contributor for the initial pulmonary injury to recruit immune cells into lung tissues. Expression of adhesion molecules such as ICAM-1 and VCAM-1 on lung vascular endothelium in HF mice is likely the outcome of increased mechanical and metabolic stress to lung vascular endothelium (31). The increase ICAM-1, VCAM-1 and MCP-1 on vascular endothelium, production of chemokines, and increased expressions of chemokine receptors and adhesion molecules on leukocytes likely contribute to the increased lung leukocyte infiltration. The accumulation of proinflammatory macrophages and lymphocytes could further damage the cardiopulmonary tissues by releasing proinflammatory cytokines and by producing reactive oxidative species. Indeed, we found that TAC-induced pulmonary inflammation and remodeling are significantly attenuated by inhibition of IL-1β signaling with blocking antibodies (32) or inhibition of oxidative stress by isolevuglandin scavenger 2-hydroxybenzylamine (2-HOBA) significantly attenuated LV failure-induced pulmonary inflammation, fibrosis, vessel remodeling, and RV hypertrophy (33). Conversely, we found that increase of lung inflammation by air pollution or pm2.5 drastically exacerbated lung fibrosis and vessel remodeling in mice with preexisting LV failure (10). The robust lung vessel muscularization and RV hypertrophy in these HF mice are consistent with the reported lung vascular remodeling and pulmonary hypertension in HF patients and in experimental HF animals (2, 28, 34), indicating the development of WHO type-2 pulmonary hypertension.

The current study supports the important role of T cells and T cell activation in TAC-induced HF development and progression (9, 11, 19). Laroumanie and associates demonstrated that HF is associated with increased cardiac T cell infiltration and activated CD4^+^ Tem increased in cardiac drainage lymph nodes in mice (11). They also demonstrated that mice lacking CD4^+^ T cells (MHCII KO) were resistant to TAC-induced LV failure, while CD8 KO did not affect TAC-induced LV failure (11). These data likely suggest that in genetic KO models, CD4 plays a bigger role in T cell development and priming and can affect multiple downstream T cell lineages, including CD8^+^ cells. However, CD8 KO models alone may not involve CD8 activation, but instead have other cell lineages compensate, such as NK cells or other immune cells. CD8 depletion does not affect T cell developmental programs, even though they may prevent tissue damage by CD8 activation in these mice. We demonstrated that HF caused a dramatic increase of CD4^+^ and CD8^+^ Tem in lung tissue, while the percentages of Tem were largely unchanged in LV tissue (9). Moreover, deficiency of CD4^+^ T cells by TCR-α KO significantly attenuated TAC-induced cardiac hypertrophy, fibrosis, and dysfunction through attenuating interferon-γ dependent cardiac fibroblast activation and proliferation (12).

Since a previous study showed that lacking CD8^+^ T cells did not affect HF development in mice (11), the finding that depletion of CD8^+^ T cells significantly attenuated HF progression is striking but was not totally unanticipated. First, CD8^+^ T cells exert a fundamental role(s) in many autoimmune diseases such as the autoimmune cardiomyopathy and immunotherapy-related myocarditis (35, 36). Therefore, CD8^+^ T cells are expected to affect the potential autoimmune responses in HF, as previous studies indicate an important role of acquired immunity in HF development and progression (11, 14, 35). The dramatic increases of the percentage of CD8^+^ Tem in lungs also suggests an important role of activated CD8^+^ T cells in the lung remodeling that occurred in the HF mice. Since CD8^+^ T cells do not cause spontaneous LV failure under normal conditions, it is anticipated that the activated CD8^+^ T cells (but not the naïve CD8^+^ T cells) promote the transition from LV failure to RV hypertrophy/failure by modulating lung inflammation. Since the activation of CD8^+^ T cells is regulated by CD4^+^ T helper cells, Tregs and antigen-presenting cells, methods to modulate these leukocyte subsets may regulate HF progression through modulating CD8^+^ T cell activation. Previously, we found that inhibition of T cell activation by either gene deletion of CD28 or B7 was highly effective in attenuating TAC-induced T cell activation and HF development (9, 14). In addition, we recently demonstrated that CD11c^+^ dendritic cells (DCs) were significantly increased in HF mice, and depletion of CD11c^+^ DCs significantly attenuated TAC-induced activation of CD4^+^ and CD8^+^ T cells, and depletion of CD11c^+^ cells also effectively attenuated TAC-induced LV hypertrophy and dysfunction in mice (14). The findings show that Treg induction suppresses lung CD8^+^ Tem infiltration while Treg depletion exacerbates lung CD8^+^ Tem infiltration in HF mice also support a central role of CD8^+^ T cells in TAC-induced HF progression. Depletion of CD8^+^ T cells was also effective in rescuing HF mice from Treg depletion-induced LV failure, lung inflammation, lung vessel remodeling, and RV hypertrophy. These findings indicate that CD8^+^ T cells also contribute to the exacerbated LV failure and lung inflammation after Treg dysfunction. The finding that mice lacking CD8^+^ T cells had no change in TAC-induced LV hypertrophy and dysfunction indicates that naïve or non-activated CD8^+^ T cells do not affect TAC-induced HF development and progression. Moreover, our findings that depletion of CD8^+^ T cells significantly attenuated TAC-induced LV hypertrophy and dysfunction clearly indicate that the reduced lung remodeling and RV hypertrophy is at least partially due to less LV failure and remodeling (such as LV inflammation and fibrosis) in these mice. Since cardiac fibrosis contribute to the diastolic dysfunction, restraining the overreactive CD8^+^ T cells may also be useful to treat cardiac diastolic dysfunction. Furthermore, since CD8^+^ T cells contribute the development of hypertension (37, 38), inhibition the overreactive CD8^+^ T cells may also protect heart through attenuating systolic hypertension in clinical setting or in rodents in response to systolic overload produced by angiotensin II or DOCA salt.

Our study demonstrates a critical role of CD8^+^ T cells in promoting pulmonary inflammation and right ventricular (RV) hypertrophy in mice with preexisting left ventricular (LV) failure. During HF development, pulmonary inflammation and remodeling exacerbate HF progression. Clarifying the role of CD8^+^ T cells in this process and targeting them for intervention could slow down HF progression. This approach offers a new perspective for treating end-stage HF.

The present study has several limitations. RV pressure was only determined in few of the experimental groups. However, since the lung vessel muscularization and RV hypertrophy are strongly correlated with RV pressure, the overall lung remodeling and HF-induced pulmonary hypertension can be effectively implied by the degree of lung vessel remodeling, lung inflammation, and RV hypertrophy in each experimental group. In addition, we only studied male mice in the present study. However, LV dysfunction, increased lung weight, lung inflammation and vascular remodeling, and RV hypertrophy are observed in both male and female mice after TAC. Since Tregs and CD8^+^ T cells respond similarly in both male and female mice, we anticipate that Tregs and CD8^+^ T cells would have similar roles in HF progression in both male and female mice. Moreover, the reduced RV hypertrophy after depletion of CD8^+^ T cells is likely a collective effect of reduced RV CD8^+^ T cell infiltration and reduced pulmonary inflammation and remodeling. Our study is unable to determine the relative impact of reduced lung remodeling and the reduced RV inflammation.

## Data Availability

The datasets presented in this study can be found in online repositories. The names of the repository/repositories and accession number(s) can be found in the article/**Supplementary Material**.
