# Supplementary figures and images for: CD8 T cells promote heart failure progression in mice with preexisting left ventricular dysfunction
Source: Front Immunol. 2024 Sep 11;15:1472133. doi: 10.3389/fimmu.2024.1472133 (PMC11422781; doi:10.3389/fimmu.2024.1472133)

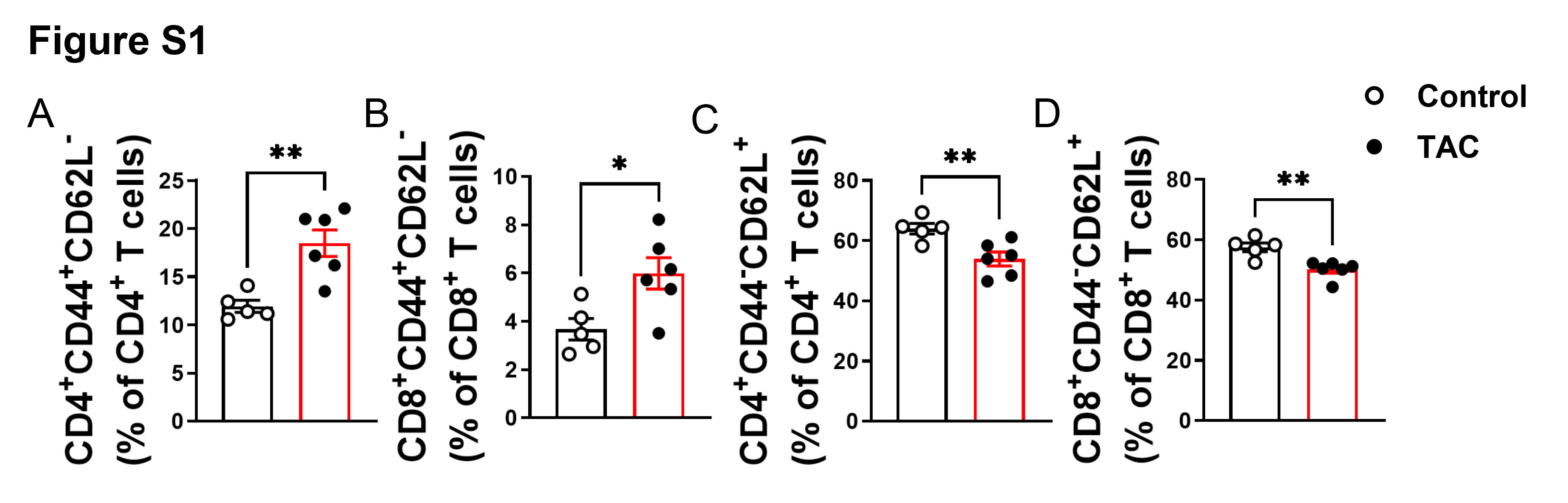

Supplement: Supplementary file 1 [file Image1.jpg]

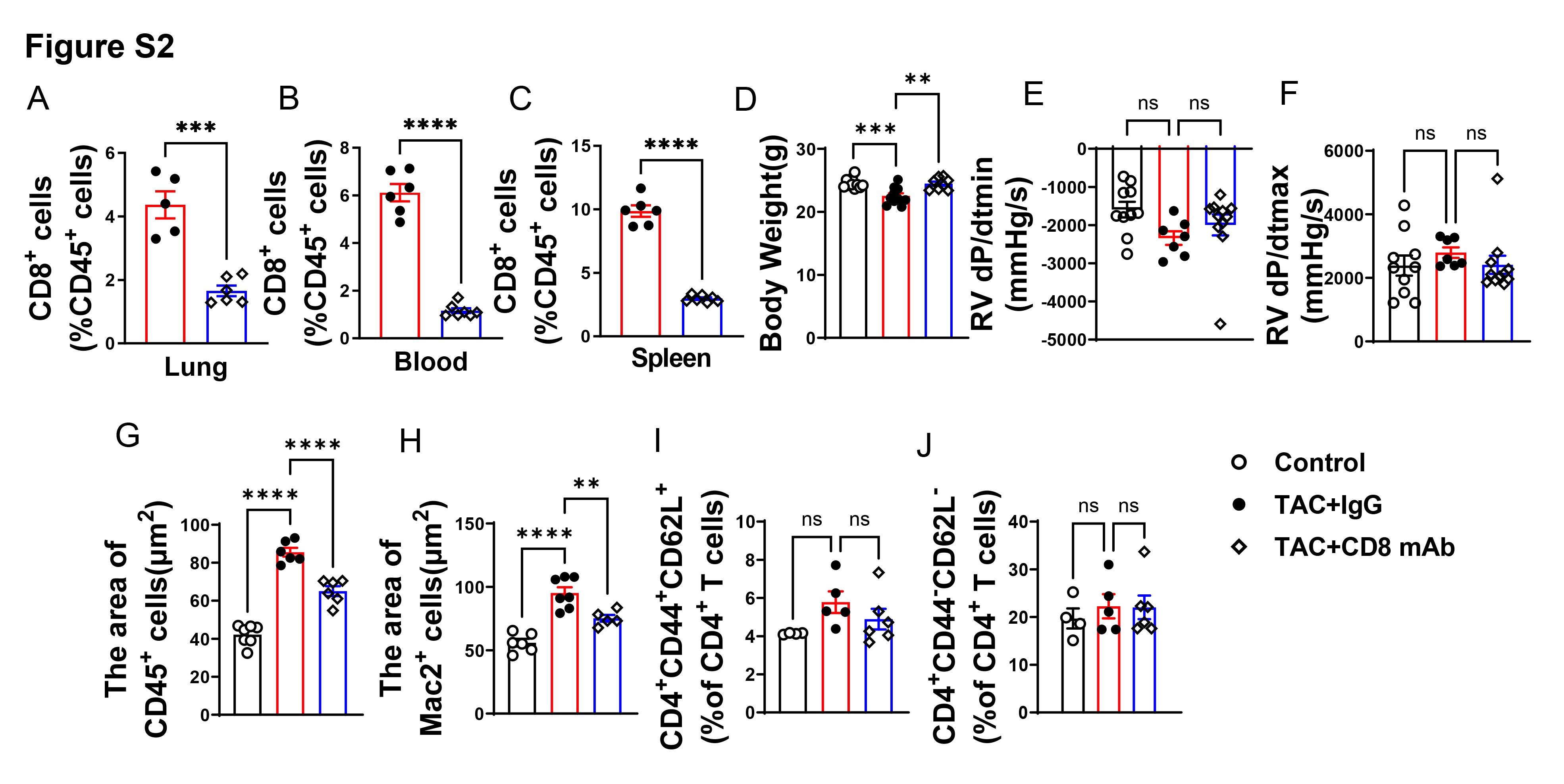

Supplement: Supplementary file 2 [file Image2.jpg]

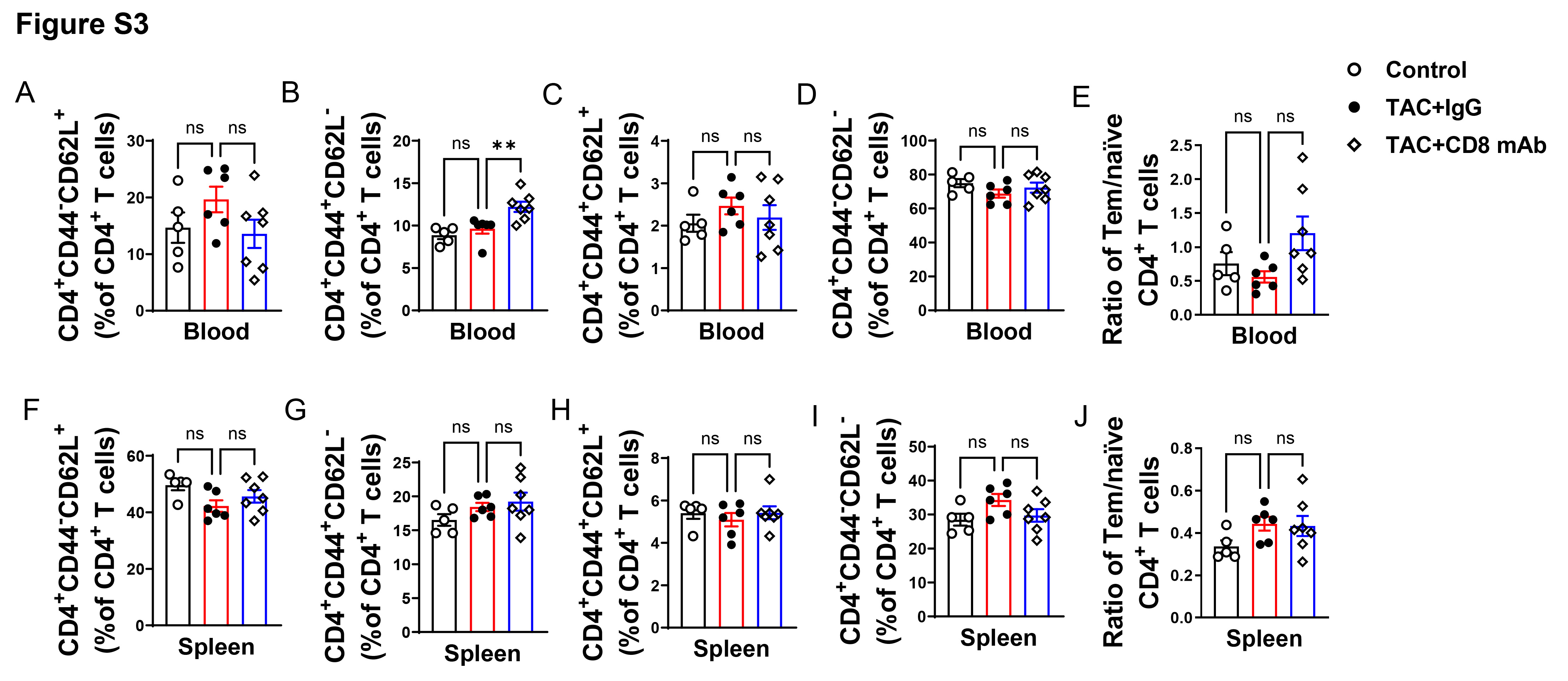

Supplement: Supplementary file 3 [file Image3.jpg]

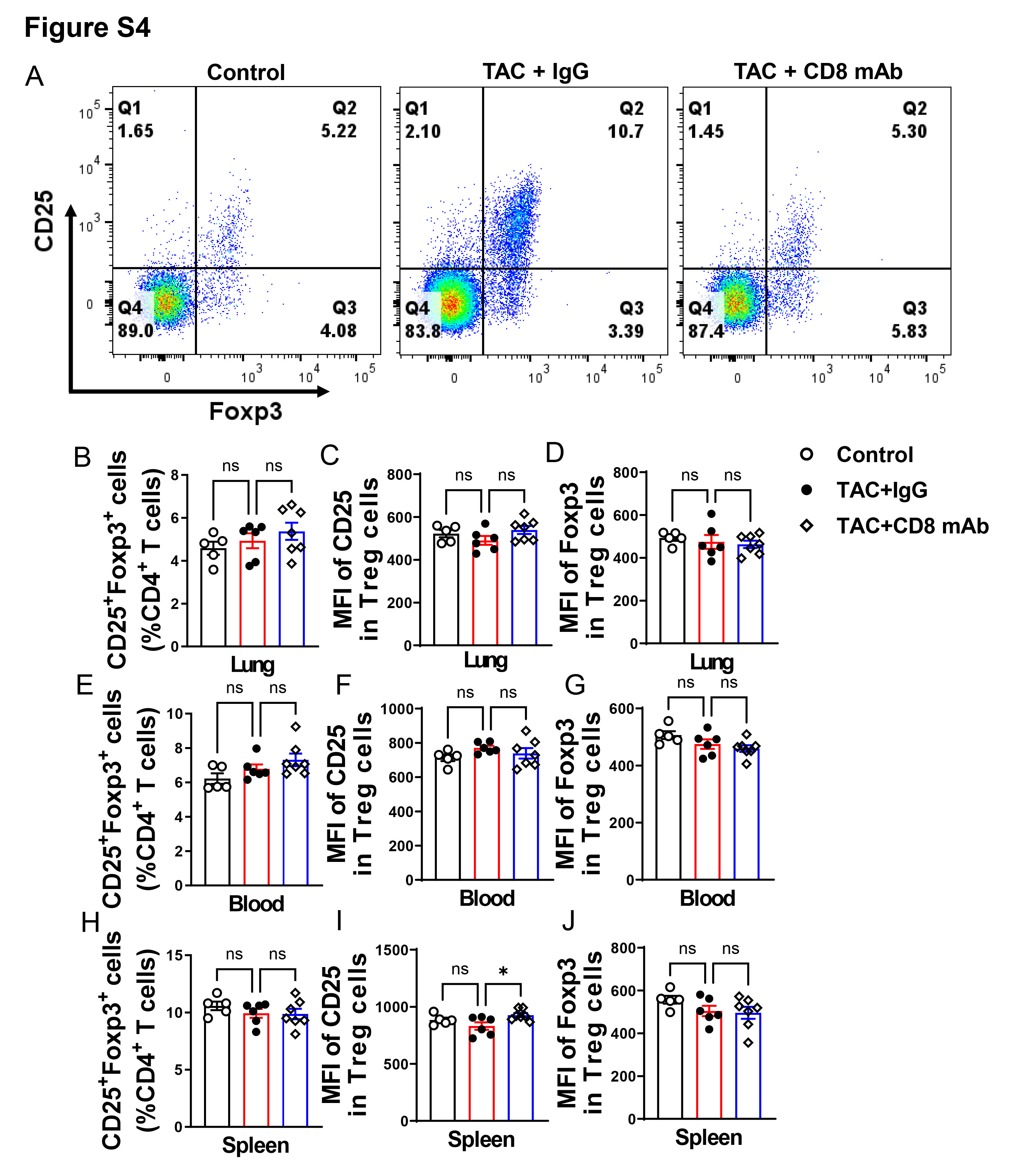

Supplement: Supplementary file 4 [file Image4.jpeg]

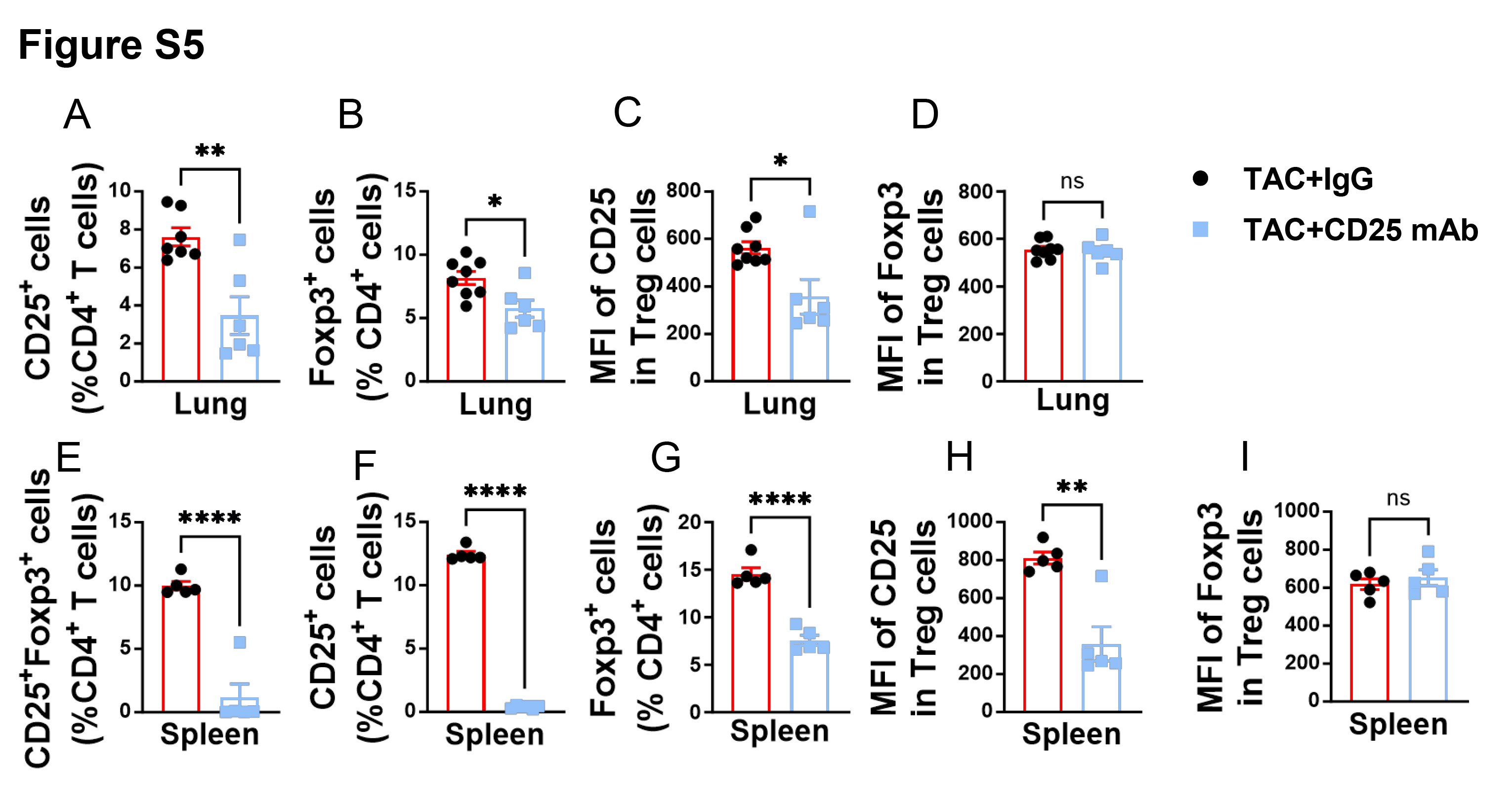

Supplement: Supplementary file 5 [file Image5.jpeg]

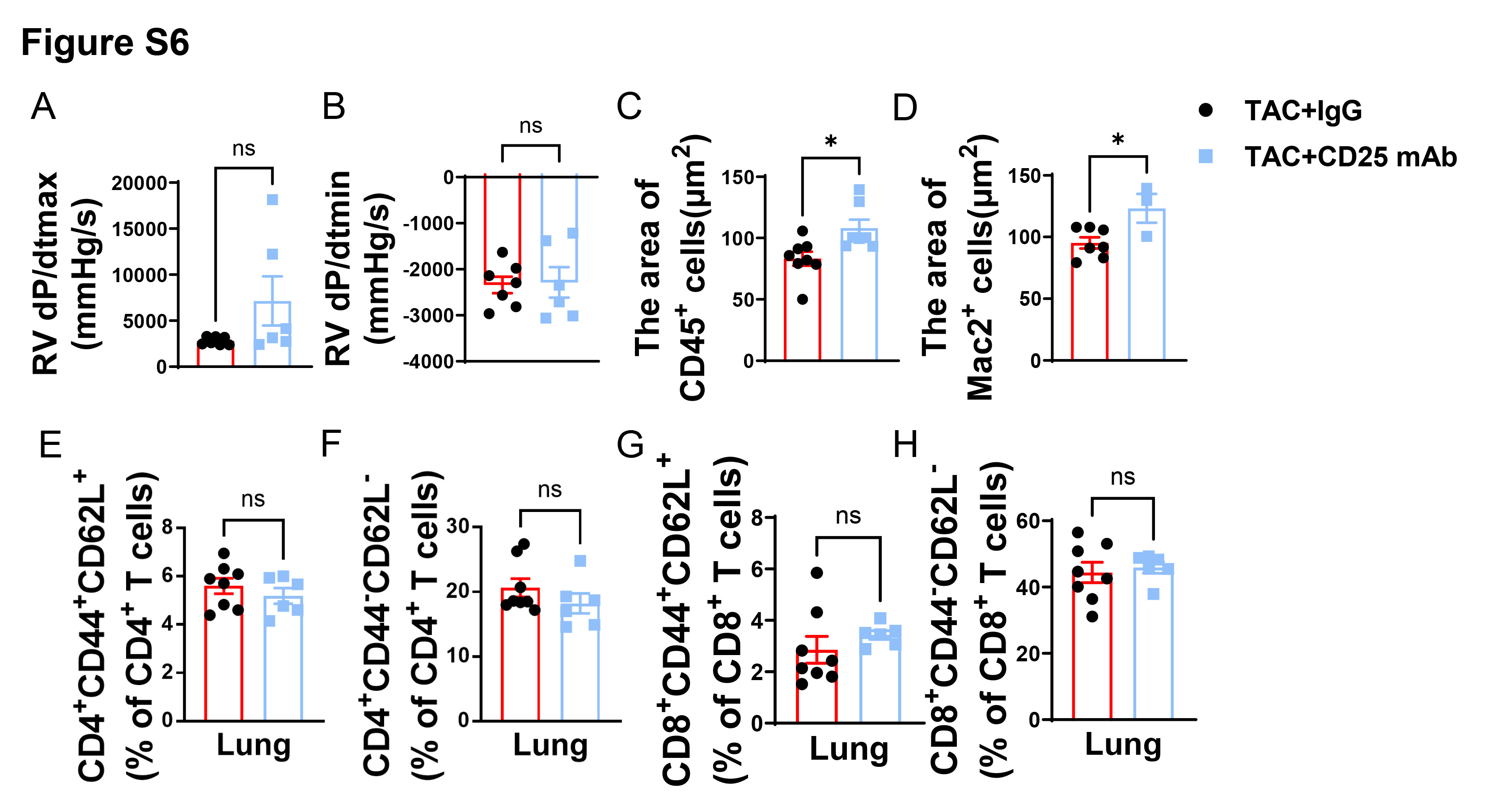

Supplement: Supplementary file 6 [file Image6.jpg]

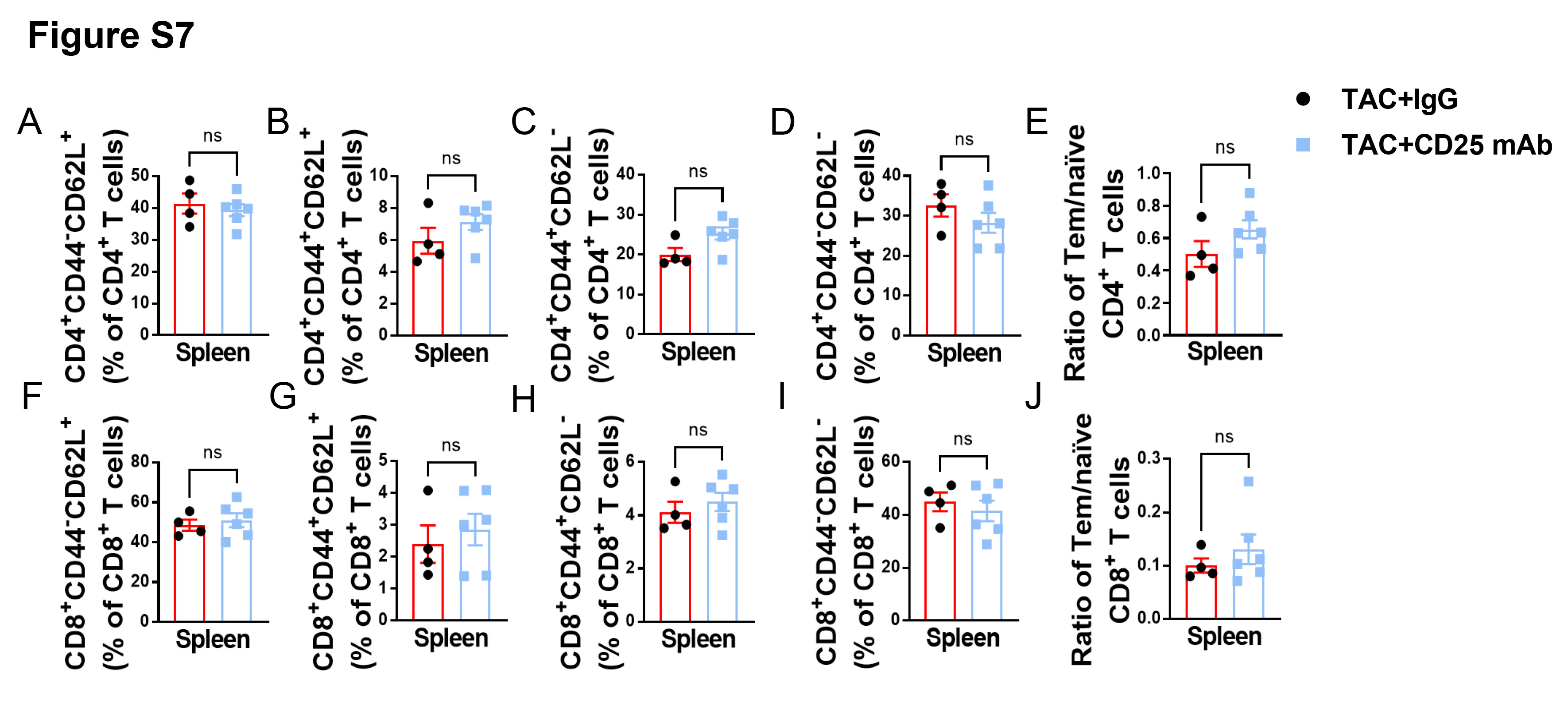

Supplement: Supplementary file 7 [file Image7.jpg]

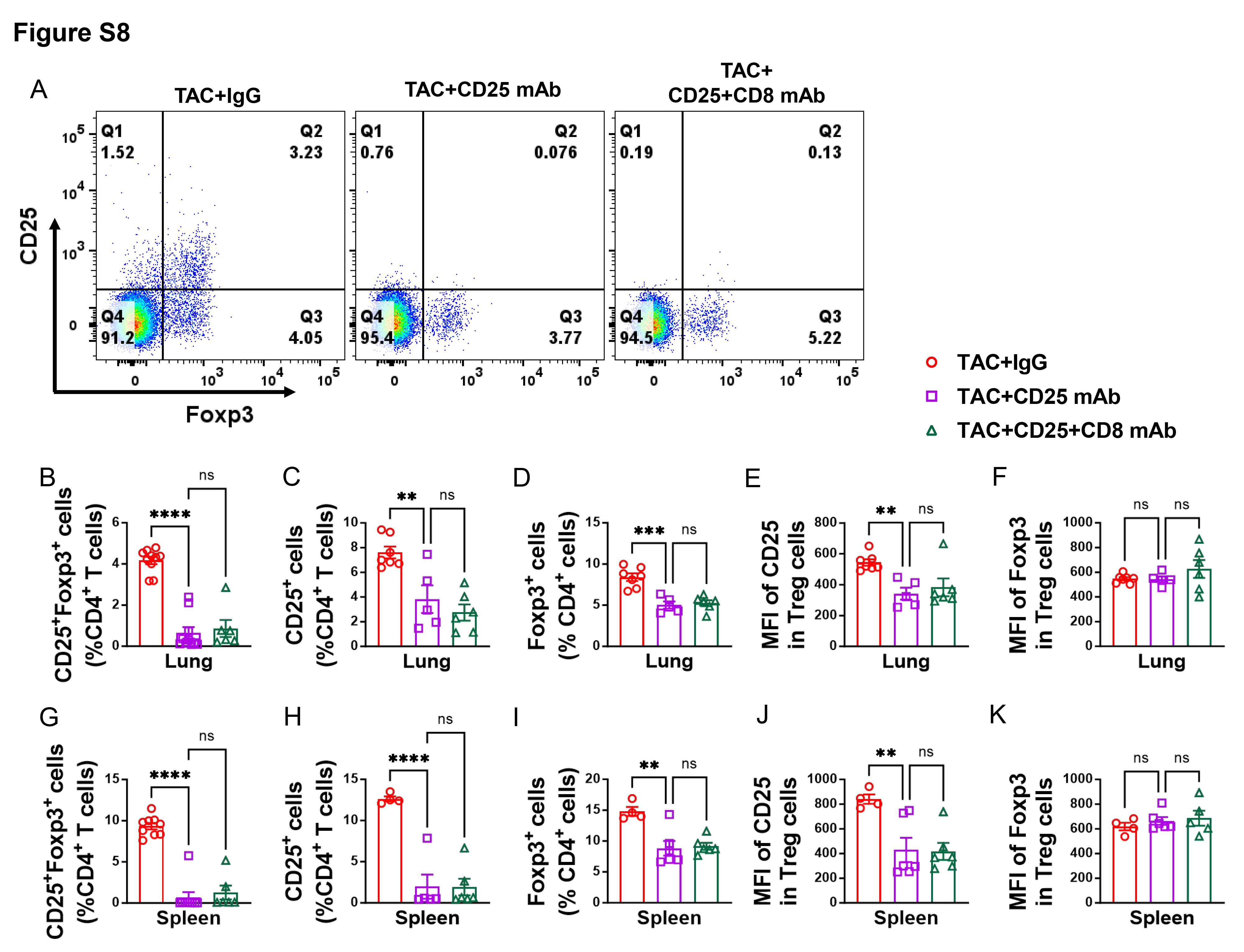

Supplement: Supplementary file 8 [file Image8.jpeg]

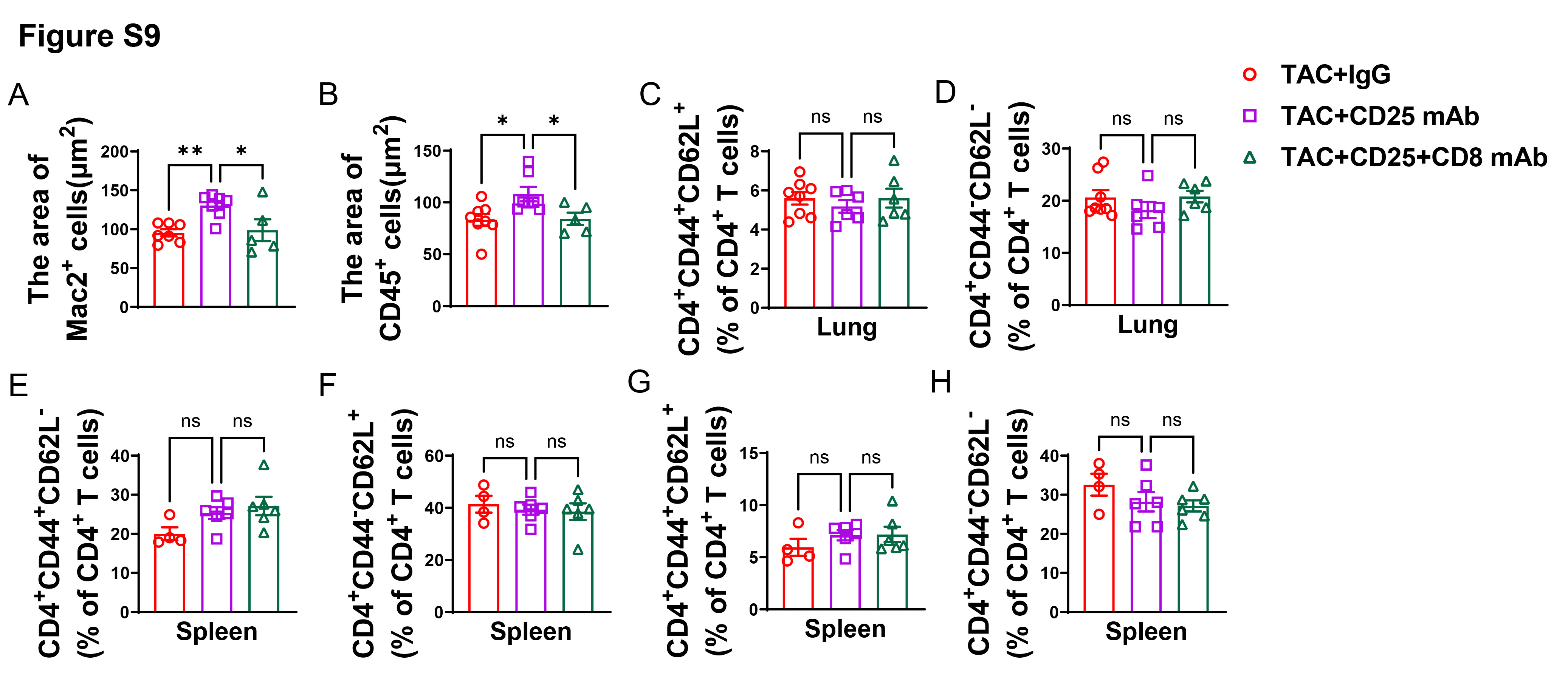

Supplement: Supplementary file 9 [file Image9.jpg]
