# Supplementary material for: CD8 T cells promote heart failure progression in mice with preexisting left ventricular dysfunction
Source: Front Immunol. 2024 Sep 11;15:1472133. doi: 10.3389/fimmu.2024.1472133 (PMC11422781; doi:10.3389/fimmu.2024.1472133)
Supplement: Supplementary file 10 [file Table1.docx]

**SUPPLEMENTAL MATERIAL**

**CD8 T cells promote heart failure progression in mice with preexisting left ventricular dysfunction**

Short title: CD8 regulates heart failure progression

Dongzhi Wang^1,2^, Ph.D.; Xinyu Weng^3,4^, Ph.D.; Wenhui Yue^2^, Ph. D; Linlin Shang^2,5^, Ph.D; Yidong Wei^2^, M.D; John S. Clemmer^1^, Ph.D. Yawei Xu^2^, MD; Yingjie Chen^1,4^, Ph.D.

^1^ Department of Physiology and Biophysics, University of Mississippi Medical center, Mississippi, Jackson, USA

^2^ Department of Cardiology, Tenth people’s hospital, Tongji University, Shanghai, China

^3^ Department of Cardiology, Zhongshan Hospital, Fudan University, Shanghai, China

^4^ Lillehei Heart Institute, University of Minnesota Medical School, Minneapolis, MN 55455, USA

^5^ Department of clinical pharmacy, School of Pharmacy, Nanjing Medical University, Nanjing, China

**Address for correspondence:**

Yingjie Chen

###### Department of Physiology and Biophysics,

###### University of Mississippi Medical Center,

###### 2500 North State Street,

###### Jackson, MS, 39216

###### Office: 601-815-3986

Email: ychen2@umc.edu

**Supplementary Materials and Methods**

**Animals model and experimental design:** Male, Balb/C wild-type (WT) mice were subjected to Transverse aortic constriction (TAC) procedure at 4-5 weeks of age with a 27G needle as previously described (1). To deplete CD8^+^ lymphocyte, 200μg anti-CD8 antibodies (Bio X cell; clone YTS 169.4) were intraperitoneally injected to the mice twice a week starting at the third week of TAC surgery for two weeks. Similarly, for depletion of regulatory T cell, 100μg anti-CD25 antibodies (Biolegend; clone PC61) were injected i.p. to the mice with a dosage of once a week starting at the third week of TAC surgery for two weeks (2). The control group was received an isotype control antibody with rat IgG. All the mice were sacrificed at the end of fourth week after TAC. Peripheral blood was collected from retinal vein. Heart, lung and spleen were collected for flow cytometry, protein and RNA analysis or histological analysis.

**Real time PCR analysis:** RNA was extracted with Trizol (Invitrogen) from mouse lung according to the manufacturer’s instructions. Quality and quantification of extracted RNA was assessed by a microplate Reader. cDNA was synthesized using TaKaRa kit. Real-time PCR was performed using KAPA kit following the manufacturer’s instructions. The primers are listed in **Table S1**. The relative amount of each gene in each sample was estimated by the ΔΔCT method. Results were normalized to 18S rRNA level.

**Histological staining**: Tissues were fixed in 10% formalin and embedded in paraffin. 5μm thick sections were used for immunostaining and 10μm thick sections were for fibrosis staining. Sections were deparaffined, rehydrated with gradient ethanol followed by antigen retrieval in Tris-EDTA buffer (PH=9.0) or citrate buffer (PH=6.0). After blocking the sections with serum at room temperature for 30 min, the primary antibodies were incubated with them together overnight at 4℃. To assess the inflammation of lung and LV, sections were incubated with primary antibody against CD45 (R&D systems, 1:100) or CD8 (abcam, 1:100) or Mac2 (Cedarlanelabs, 1:1000). Different florescence conjugated secondary antibodies were used to manifest the results. Alexa Fluor 555-conjugated goat anti-rat antibody (1:500) (Yeasen, Shanghai) was used as secondary antibody for Mac2, Alexa Fluor 555-conjugated goat anti-rabbit antibody (1:500) (Yeasen, Shanghai) was the secondary antibody for CD8, and Alexa Fluor 555-conjugated donkey anti-goat antibody (1:500) (Yeasen, Shanghai) was for CD45. The remodeling of pulmonary vascular was evaluated by double immunofluorescence staining of CD31 (eBscience, 1:100) with α-smooth muscle actin (1:500) (α-actin, Santa Cruz), Alexa Fluor 448-conjugated goat anti-hamster antibody (1:500) was the secondary antibody for CD31, and Alexa Fluor 555-conjugated goat anti-mouse (1:500) was for α-smooth muscle actin, respectively. DAPI was contained within mounting medium. The images were captured using a ZEISS confocal microscope (FluoView 1000 Olympus). CD8, CD45 and mac2 positive cells were quantified by image J. According to the SMA around the vessel, vessels with a diameter of 10 to 50μm were calculated and categorized as non-muscularized (NM, <25%), partially muscularized (PM, 25% to 75%), or fully muscularized (FM, >75%) (3). The relative percentage of NM, PM and FM is calculated as each muscularization category/ the total number of the vessels. For RV cardiomyocyte size evaluation, FITC-conjugated wheat germ agglutinin (AF488, Sigma) was used, the cross-sectional area was quantified with image J software, at least 120 cells/sample (from 5 areas) and at least 5 samples of each group were averaged. Sirius red and fast green Stain Kit (Chondrex, Inc) was used to assess the fibrosis of RV, and lung fibrosis was stained using Masson trichrome kit. Fibrosis quantification was calculated by image J. Samples from at least 5 mice were analyzed per group.

**Statistics:** A normality test (Shapiro-Wilk) provided by SPSS was used to determine whether data were normally distributed. If data were normally distributed, the data were presented as mean ± SEM. A Student’s t-test was used to test for differences between 2 groups. A one-way ANOVA followed by a Bonferroni correction post-hoc test was used to test for differences among more than 2 groups. If mouse physiological data were not normally distributed or the sample size in one of the experimental groups was less than 10, a non-parametric test (Mann-Whitney or Kruskal-Wallis) followed by a Bonferroni post hoc correction was performed. All pairwise p-values are two-sided. The null hypothesis was rejected at P < 0.05.

**References**

1. Wang H, Kwak D, Fassett J et al. CD28/B7 Deficiency Attenuates Systolic Overload-Induced Congestive Heart Failure, Myocardial and Pulmonary Inflammation, and Activated T Cell Accumulation in the Heart and Lungs. Hypertension. (2016)68:688-96. doi: 10.1161/HYPERTENSIONAHA.116.07579.

2. Ueha, S. Yokochi, Y. Ishiwata, H. Ogiwara, K. Chand, T. Nakajima, et al. Cancer Immunol Res. (2015) 3:631-40. doi: 10.1158/2326-6066.CIR-14-0190.

3. Soni Savai Pullamsetti BK, Samantha Storn, et al. Lung cancer–associated pulmonary hypertension: Role of microenvironmental inflammation based on tumor cell–immune cell cross-talk. Sci Transl Med. (2017)9: eaai9048. doi:[10.1126/scitranslmed.aai9048](https://doi.org/10.1126/scitranslmed.aai9048).

**Table S1. Primers used in quantitative real-time PCR.**

| **Mouse Gene** | | | |
| --- | --- | --- | --- |
| **TNF-α** | **sense** | 5’-ATGTCTCAGCCTCTTCTCATTC-3’ |  |
|  | **antisense** | 5’-GCTTGTCACTCGAATTTTGAGA-3’ |  |
| **TGF-β** | **sense** | 5’-CCT GAG TGG CTG TCT TTT GA-3’ |  |
|  | **antisense** | 5’-CGT GGA GTT TGT TAT CTT TGC TG-3’ |  |
| **MCP-1** | **sense** | 5’-TTTTTGTCACCAAGCTCAAGAG-3’ |  |
|  | **antisense** | 5’-TTCTGATCTCATTTGGTTCCGA-3’ |  |
| **Collagen III** | **sense** | 5’-TCCCCTGGAATCTGTGAATC-3’ |  |
|  | **antisense** | 5’-TGAGTCGAATTGGGGAGAAT-3’ |  |
| **CXCL9** | **sense** | 5’-GGAGTTCGAGGAACCCTAGTG-3’ |  |
|  | **antisense** | 5’-GGGATTTGTAGTGGATCGTGC-3’ |  |
| **CXCL10** | **sense** | 5’-CCAAGTGCTGCCGTCATTTTC-3’ |  |
|  | **antisense** | 5’-GGCTCGCAGGGATGATTTCAA-3’ |  |
| **CXCL11** | **sense** | 5’-TGTAATTTACCCGAGTAACGGC-3’ |  |
|  | **antisense** | 5’-CACCTTTGTCGTTTATGAGCCTT |  |
| **18s** | **sense** | 5’-GCAATTATTCCCCATGAACG-3’ |  |
|  | **antisense** | 5’-GGCCTCACTAAACCATCCAA-3’ |  |
|  | | | |

**Table S2. Primary antibodies used in our study.**

| **Antibody** | **Clone** | **Name of the company** | **Catalog number** |
| --- | --- | --- | --- |
| **anti-mouse CD16/32** | 2.4G2 | BD Biosciences | 553142 |
| **APC/Cy7-conjugated anti-CD45** | 30-F11 | BD Biosciences | 557659 |
| **FITC-conjugated anti-CD4** | GK1.5 | BD Biosciences | 557307 |
| **PerCP/Cy5.5-conjugated anti-CD8** | 53-6.7 | BD Biosciences | 551162 |
| **PE-conjugated anti-CD44** | IM7 | BD Biosciences | 553134 |
| **APC-conjugated anti-CD62L** | MEL-14 | BD Biosciences | 553152 |
| **APC-conjugated anti-CD25** | PC61 | BD Biosciences | 557192 |
| **PE-conjugated anti-Foxp3** | FJK-16s | eBioscience | 12-5773 |
| **CD45** |  | R&D systems | AF114 |
| **Mac2** |  | Cedarlanelabs | CL8942AP |
| **CD8** | YTS 169AG 101HL | abcam | ab112219 |
| **CD31** | 2H8 | Millipore | MAB1398Z |
| **α-Actin** |  | Santa cruz | sc-32251 |
| **InvivoMAb CD8α** | YTS 169.4 | Bioxcell | BE0117 |
| **InvivoMAb CD25** | PC61 | Biolegend | 102040 |

**Table S3. Anatomic data of mice with LV failure treated with CD8 mAb or IgG**

| **Parameters** | **Control** | **TAC+IgG** | **TAC+CD8 mAb** |
| --- | --- | --- | --- |
| **Number of mice** | 8 | 10 | 8 |
| **Bodyweight (g)** | 24.3±0.19 | 22.9±0.58* | 24.5±0.34# |
| **Left ventricular (LV) weight (mg)** | 80.8±1.33 | 126±3.47* | 113±1.81# |
| **Left atria (LA) weight (mg)** | 3.4±0.19 | 18.2±3.25* | 7.0±0.62# |
| **Lung mass (mg)** | 145±7.73 | 324±21.56* | 199±26.16# |
| **Right ventricular (RV) weight (mg)** | 20.2±0.64 | 23.7±0.74* | 21.4±0.57# |
| **Ratio of LV weight to body weight (mg/g)** | 3.33±0.07 | 5.50±0.24* | 4.64±0.11# |
| **Ratio of LA weight to body weight (mg/g)** | 0.14±0.02 | 0.81±0.15* | 0.28±0.02# |
| **Ratio of lung weight to body weight (mg/g)** | 6.0±0.31 | 14.2±0.99* | 8.14±1.07# |
| **Ratio of RV weight to body weight (mg/g)** | 0.83±0.02 | 1.04±0.05* | 0.87±0.02# |
| **Tibial length (mm)** | 17.4±0.09 | 16.9±0.18* | 17.0±0.12# |
| **Ratio of LV weight to tibial length (mg/mm)** | 4.66±0.09 | 7.42±0.19* | 6.66±0.11# |
| **Ratio of LA weight to tibial length (mg/mm)** | 0.20±0.01 | 1.05±0.19* | 0.41±0.04# |
| **Ratio of lung weight to tibial length (mg/mm)** | 8.38±0.46 | 19.1±1.16* | 11.7±1.60# |
| **Ratio of RV weight to tibial length (mg/mm)** | 1.17±0.04 | 1.40±0.04* | 1.25±0.11# |

Data are presented as mean ± SEM. *p<0.05 as compared with corresponding control group. #p<0.05 as compared with corresponding TAC+IgG conditions.

**Table S4. Anatomic data of mice with LV failure treated with CD25 mAb or IgG**

| **Parameters** | **TAC+IgG** | **TAC+CD25 mAb** |
| --- | --- | --- |
| **Number of mice** | 8 | 6 |
| **Bodyweight (g)** | 21.5±0.48 | 18.8±0.20* |
| **Left ventricular (LV) weight (mg)** | 118±4.47 | 129±3.48 |
| **Left atria (LA) weight (mg)** | 15.5±1.42 | 17.7±1.45 |
| **Lung mass (mg)** | 284±25.84 | 454±17.97* |
| **Right ventricular (RV) weight (mg)** | 22.8±1.05 | 25.8±1.05* |
| **Ratio of LV weight to body weight (mg/g)** | 5.50±0.23 | 6.84±0.17* |
| **Ratio of LA weight to body weight (mg/g)** | 0.73±0.07 | 0.94±0.07 |
| **Ratio of lung weight to body weight (mg/g)** | 13.2±1.02 | 24.1±2.11* |
| **Ratio of RV weight to body weight (mg/g)** | 1.06±0.04 | 1.37±0.05* |
| **Tibial length (mm)** | 16.9±0.26 | 16.8±0.14 |
| **Ratio of LV weight to tibial length (mg/mm)** | 6.99±0.26 | 7.66±0.24 |
| **Ratio of LA weight to tibial length (mg/mm)** | 0.92±0.08 | 1.05±0.09 |
| **Ratio of lung weight to totibial length (mg/mm)** | 16.8±1.38 | 27.0±1.02* |
| **Ratio of RV weight to body weight (mg/g)** | 1.35±0.04 | 1.54±0.06* |

Data are presented as mean ± SEM. *p<0.05 as compared with corresponding TAC + IgG group.

**Table S5. Anatomic data of mice with LV failure treated with CD8 mAb or CD25 mAb**

| **Parameters** | **TAC+IgG** | **TAC+CD25 mAb** | **TAC+CD8+CD25 mAb** |
| --- | --- | --- | --- |
| **Number of mice** | 10 | 12 | 7 |
| **Bodyweight (g)** | 21.3±0.31 | 19.0±0.22* | 20.9±0.51# |
| **Left ventricular (LV) weight (mg)** | 115±3.92 | 123±3.05 | 105±4.21# |
| **Left atria (LA) weight (mg)** | 14.1±1.87 | 26.4±3.17* | 12.1±1.81# |
| **Lung mass (mg)** | 232±22.30 | 420±20.49* | 246±23.65# |
| **Right ventricular (RV) weight (mg)** | 21.3±0.68 | 24.7±0.76* | 19.3±0.85# |
| **Ratio of LV weight to body weight (mg/g)** | 5.41±0.21 | 6.49±0.19* | 5.03±0.20# |
| **Ratio of LA weight to body weight (mg/g)** | 0.60±0.10 | 1.39±0.17* | 0.59±0.10# |
| **Ratio of lung weight to body weight (mg/g)** | 11.0±1.09 | 22.2±1.20* | 11.9±1.27# |
| **Ratio of RV weight to body weight (mg/g)** | 1.00±0.04 | 1.30±0.04* | 0.93±0.04# |
| **Tibial length (mm)** | 16.6±0.20 | 16.9±0.09 | 16.7±0.26 |
| **Ratio of LV weight to tibial length (mg/mm)** | 6.94±0.22 | 7.31±0.19 | 6.30±0.30# |
| **Ratio of LA weight to tibial length (mg/mm)** | 0.77±0.13 | 1.56±0.19* | 0.73±0.12# |
| **Ratio of lung weight to tibial length (mg/mm)** | 13.9±1.27 | 24.9±1.26* | 14.8±1.50# |
| **Ratio of RV weight to tibial length (mg/mm)** | 1.28±0.03 | 1.46±0.04* | 1.16±0.05# |

Data are presented as mean±SEM. *p<0.05 as compared with corresponding TAC+IgG group. #p<0.05 as compared with corresponding TAC+CD25 mAb group.

**Figure Caption**

**Figure S1**. **LV failure caused lung T cell activation in mice. (A), (B),** Percentage of CD4^+^CD44^+^CD62L^-^ and CD8^+^CD44^+^CD62L^-^ activated T cells in lung. **(C), (D),** Percentage of CD4^+^CD44^-^CD62L^+^ and CD8^+^CD44^-^CD62L^+^ naïve T cells in lung. Error bars represent SEM; *P<0.05; **P<0.01; ***P<0.001, ns, non-significant.

**Figure S2**. **CD8 mAb treatment** **attenuates lung inflammation in mice with existing LV failure.** **(A)-(C),** Quantitative data of CD8^+^ T cells in lung, blood and spleen. **(D),** Body weight of mice. **E,** Right Ventricular dp/dt min(mmHg/s). **(F),** Right Ventricular dp/dt max(mmHg/s). **(G), (H),** Quantitative data of the area of CD45^+^ cells and Mac2^+^ cells. **(I), (J),** Percentage of CD4^+^CD44^+^CD62L^+^central memorial T cells and CD4^+^CD44^-^ CD62L^-^ T cells in lung. Error bars represent SEM; *P<0.05; **P<0.01; ***P<0.001, ns, non-significant.

**Figure S3**. **CD8 mAb treatment doesn’t change T cells activation of blood and spleen in mice with existing LV failure. (A)-(D),** Percentage of CD4^+^CD44^-^CD62L^+^naïve T cells, CD4^+^CD44^+^CD62L^-^ Tem, CD4^+^CD44^+^CD62L^+^central memorial T cells and CD4^+^CD44^-^ CD62L^-^ T cells in blood. **(F)-(I),** Percentage of CD4^+^CD44^-^CD62L^+^naïve T cells, CD4^+^CD44^+^CD62L^-^ Tem, CD4^+^CD44^+^CD62L^+^central memorial T cells and CD4^+^CD44^-^ CD62L^-^ T cells in spleen. **(E), (J),** Ratio of Tem to naïve T cells in blood and spleen. Error bars represent SEM; *P<0.05; **P<0.01; ***P<0.001, ns, non-significant.

**Figure S4**. **CD8 mAb treatment doesn’t change Treg of lung, blood and spleen in mice with existing LV failure. (A),** Scatterplots representing percentages of Treg cells in lung. **(B),** Percentage of CD4^+^CD25^+^Foxp3^+^Treg in lung. **(C), (D),** Flow cytometric analysis of lung tissue indicating mean fluorescence intensity (MFI) of CD25 and Foxp3 on Treg. **(E),** Percentage of CD4^+^CD25^+^Foxp3^+^Treg in blood. **(F), (G),** Flow cytometric analysis of blood indicating mean fluorescence intensity (MFI) of CD25 and Foxp3 on Treg. **(H),** Percentage of CD4^+^CD25^+^Foxp3^+^Treg in spleen. **(I), (J),** Flow cytometric analysis of spleen indicating mean fluorescence intensity (MFI) of CD25 and Foxp3 on Treg. Error bars represent SEM; *P<0.05; **P<0.01; ***P<0.001, ns, non-significant.

**Figure S5**. **CD25 mAb was effective in depletion of Tregs in mice with existing LV failure. (A), (B),** Percentage of CD4^+^CD25^+^ T cells and CD4^+^Foxp3^+^ T cells in lung. **(C), (D),** Flow cytometric analysis of lung indicating mean fluorescence intensity (MFI) of CD25 and Foxp3 on Treg. **(E), (F), (G),** Percentage of CD4^+^CD25^+^Foxp3^+^Treg, CD4^+^CD25^+^ T cells and CD4^+^Foxp3^+^ T cells in spleen. **(H), (I),** Flow cytometric analysis of lung indicating mean fluorescence intensity (MFI) of CD25 and Foxp3 on Treg. Error bars represent SEM; *P<0.05; **P<0.01; ***P<0.001, ns, non-significant.

**Figure S6. Treg depletion increased the leukocyte infiltration in mice with existing LV failure. (A),** Right Ventricular dp/dt min(mmHg/s). **(B),** Right Ventricular dp/dt max(mmHg/s). **(C), (D),** Quantitative data of the area of CD45^+^ cells and Mac2^+^ cells in lung. **(E), (F),** Percentage of CD4^+^CD44^+^CD62L^+^central memorial T cells and CD4^+^CD44^-^ CD62L^-^ T cells in lung. **(G), (H),** Percentage of CD8^+^CD44^+^CD62L^+^central memorial T cells and CD8^+^CD44^-^ CD62L^-^ T cells in lung. Error bars represent SEM; *P<0.05; **P<0.01; ***P<0.001, ns, non-significant.

**Figure S7**. **Treg depletion didn’t change T cell activation of spleen in mice with existing LV failure. (A)-(D),** Percentage of CD4^+^CD44^-^CD62L^+^ naïve T cells, CD4^+^CD44^+^CD62L^+^central memorial T cells, CD4^+^CD44^+^CD62L^-^ Tem and CD4^+^CD44^-^CD62L^-^ T cells in spleen. **(F)-(I),** Percentage of CD8^+^CD44^-^CD62L^+^naïve T cells, CD8^+^CD44^+^CD62L^+^central memorial T cells, CD8^+^CD44^+^CD62L^-^ Tem and CD8^+^CD44^-^ CD62L^-^ T cells in spleen. **(E), (J),** Ratio of Tem to naïve T cells in spleen. Error bars represent SEM; *P<0.05; **P<0.01; ***P<0.001, ns, non-significant.

**Figure S8. Depletion of CD8^+^ T cells did not affect Treg depletion caused by CD25 mAb blocking. (A),** Scatterplots representing percentages of Treg cells in lung. **(B)-(D),** Percentage of CD4^+^CD25^+^Foxp3^+^Treg, CD4^+^CD25^+^ T cells and CD4^+^Foxp3^+^ T cells in lung. **(E), (F),** Flow cytometric analysis of lung tissue indicating mean fluorescence intensity (MFI) of CD25 and Foxp3 on Treg. **(G)-(I),** Percentage of CD4^+^CD25^+^Foxp3^+^Treg, CD4^+^CD25^+^ T cells and CD4^+^Foxp3^+^ T cells in spleen. **(J), (K),** Flow cytometric analysis of spleen tissue indicating mean fluorescence intensity (MFI) of CD25 and Foxp3 on Treg. Error bars represent SEM; *P<0.05; **P<0.01; ***P<0.001, ns, non-significant.

**Figure S9**. **Treatment with CD8 mAb rescued the aggravation of lung leukocyte infiltration induced by Treg depletion in mice with existing LV failure, without affecting the spleen. (A), (B),** Quantitative data of the area of CD45^+^ cells and Mac2^+^ cells in lung. **(C), (D),** Percentage of CD4^+^CD44^+^CD62L^+^central memorial T cells and CD4^+^CD44^-^ CD62L^-^ T cells in lung. **(E)-(F),** Percentage of CD4^+^CD44^+^CD62L^-^ Tem, CD4^+^CD44^-^CD62L^+^naïve T cells, CD4^+^CD44^+^CD62L^+^central memorial T cells and CD4^+^CD44^-^ CD62L^-^ T cells in spleen. Error bars represent SEM; *P<0.05; **P<0.01; ***P<0.001, ns, non-significant.
